# Noise-injected neural networks show promise for use on small-sample expression data

**DOI:** 10.1186/1471-2105-7-274

**Published:** 2006-05-31

**Authors:** Jianping Hua, James Lowey, Zixiang Xiong, Edward R Dougherty

**Affiliations:** 1 Computational Biology Division, Translational Genomics Research Institute, Phoenix, USA; 2 Department of Electrical and Computer Engineering, Texas A&M University, College Station, USA

## Abstract

**Background:**

Overfitting the data is a salient issue for classifier design in small-sample settings. This is why selecting a classifier from a constrained family of classifiers, ones that do not possess the potential to too finely partition the feature space, is typically preferable. But overfitting is not merely a consequence of the classifier family; it is highly dependent on the classification rule used to design a classifier from the sample data. Thus, it is possible to consider families that are rather complex but for which there are classification rules that perform well for small samples. Such classification rules can be advantageous because they facilitate satisfactory classification when the class-conditional distributions are not easily separated and the sample is not large. Here we consider neural networks, from the perspectives of classical design based solely on the sample data and from noise-injection-based design.

**Results:**

This paper provides an extensive simulation-based comparative study of noise-injected neural-network design. It considers a number of different feature-label models across various small sample sizes using varying amounts of noise injection. Besides comparing noise-injected neural-network design to classical neural-network design, the paper compares it to a number of other classification rules. Our particular interest is with the use of microarray data for expression-based classification for diagnosis and prognosis. To that end, we consider noise-injected neural-network design as it relates to a study of survivability of breast cancer patients.

**Conclusion:**

The conclusion is that in many instances noise-injected neural network design is superior to the other tested methods, and in almost all cases it does not perform substantially worse than the best of the other methods. Since the amount of noise injected is consequential, the effect of differing amounts of injected noise must be considered.

## Background

### Classifier complexity and overfitting

The small-sample problems with microarray-based classification have long been recognized [1]. The potential number of features (variables) upon which a classifier can be based is extremely large, the potential features consisting of all the gene-expression levels measured on a microarray (20,000 or more), and the sample size being the number of microarrays in the study (usually less than 100 and often less than 50). When the number of features is large in comparison to the sample size, classifier design is hampered by the designed classifier tending to overfit the sample data, which means that the designed classifier may provide good discrimination for the sample data but not for the general population from which the sample data have been drawn.

Classifier design involves choosing a classifier from a family of classifiers on the basis of the data by means of some algorithm. In this paper we restrict our attention to the case of two classes. Classification involves a *feature vector ***X **= (*X*_1_, *X*_2_,..., *X*_*d*_) on *d*-dimensional Euclidean space ℜ^*d *^composed of random variables (*features*), a binary random variable *Y*, and a function (*classifier*) ψ: ℜ^*d *^→ {0, 1} to serve as a predictor of *Y*. The values, 0 or 1, of *Y *are treated as class *labels*. The error of ψ is the probability, *P*(ψ(**X**) ≠ *Y*), that the classification is erroneous. Classifier error depends on the probability distribution, *f*_**x**, *y*_(**x**, *y*), called the *feature-label distribution*, of the feature-label pair (**X**, *Y*), in particular, the *class conditional distributions*, *f*_**x**|0_(**x**) and *f*_**x**|1_(**x**). Since in practice we do not know the class conditional distributions, a classifier is designed from sample data.

A classifier is optimal in a family *G *of classifiers if its error, ε_*G*_, is minimal among all classifiers in *G*. Since a designed classifier depends on the particular sample, it is random relative to random sampling. We would like the expected error, ε_*G*, *n*_, of the designed classifier, *n *denoting sample size, to be close to ε_*G*_. If *G *and *H *are families of classifiers such that *G *⊂ *H*, then ε_*H *_≤ ε_*G*_; however, for designed classifiers, it may be that ε_*H*, *n *_> ε_*G*, *n*_. That is, the designed classifier may partition the feature space well relative to the sample data but not relative to the full distribution. This phenomenon, called *overfitting*, is widespread in studies with small sample sizes. To mitigate overfitting, one can choose from smaller classifier families whose classifiers partition the feature space more coarsely. Using *G *instead of *H*, where *G *⊂ *H*, reduces the *design cost*, ε_*G*, *n *_- ε_*G*_, relative to ε_*H*, *n *_- ε_*H *_at the expense of introducing a *constraint cost*, ε_*G *_- ε_*H*_. For a fixed sample size *n*, consider a collection of classifier families, *G*_1 _⊂ *G*_2 _⊂ *G*_3 _⊂ .... The increasing size of the families means increasing classifier complexity. While the smaller families extensively reduce design cost, their constraint is excessive thus creating a situation in which the expected errors of the designed classifiers fall as we utilize increasingly large families but then begin to increase when the design cost grows too much.

While overfitting is often thought of as applying to the complexity of functional structure of a classifier, it also applies to the number of features composing a classifier. A feed-forward neural network classifier with one hidden layer, *d *features and *k *hidden nodes, is an operator on *d *dimensional Euclidean space ℜ^*d*^, as is a linear classifier. Their difference in complexity is that the linear classifier partitions ℜ^*d *^into two classes via a hyperplane, whereas the neural net more finely partitions the space, thereby reflecting greater complexity. Another way to increase complexity is to increase the number of features. In this way a linear classifier on *d *+ 1 features is more complex than a linear classifier on *d *variables because the former reduces to the latter by setting one of the variables equal to 0. In this vein, if we consider a sequence, *x*_1_, *x*_2_, ..., *x*_*d*_, ..., of features, we often first observe a decrease in expected error as *d *increases and then subsequently an increase in error for increasing *d*. While this description is idealized and the situation can be more complex, it describes the *peaking phenomenon*.

**Figure 1:**
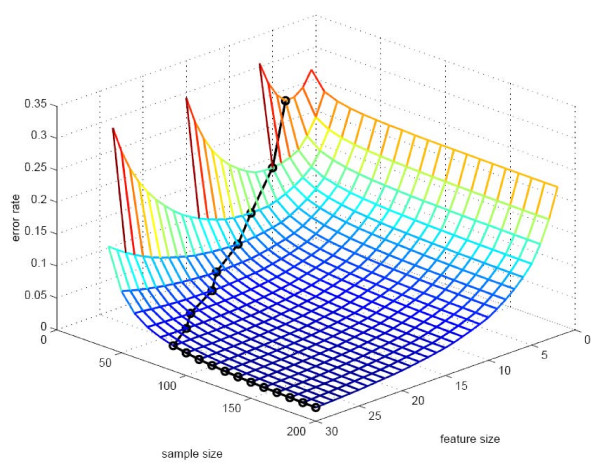
Peaking phenomenon for LDA with slightly correlated features.

**Figure 2:**
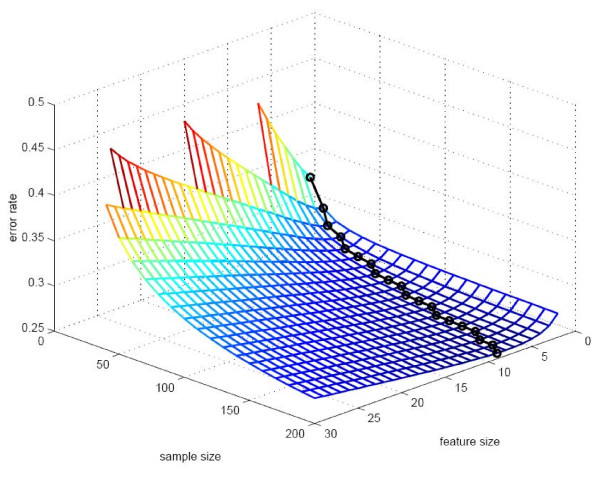
Peaking phenomenon for LDA with highly correlated features.

In light of the peaking phenomenon, a natural question arises: given a set of potential features, what is the optimal number of features one should use for classifier design [2]? The question is complicated because it depends on the classification rule, feature-label distribution and sample size. Figure 1 illustrates peaking in terms of sample size *n *and the number *d *of features. The surface gives the average error of designed LDA classifiers in terms of *d *and *n *based on two Gaussian class conditional distributions possessing the same covariance matrix. The features are slightly correlated and we see that peaking occurs with very few features for sample sizes 30 and below, but then exceeds 30 features for sample sizes above 90. A much more serious situation for LDA is presented in Fig. 2, where the situation is the same except that the features are highly correlated. With a sample size of 80, large for most microarray studies, the optimal number of features is 3. Even with a sample size of 200, the optimal number of features is only 8.

In our preceding examples, we knew the distributions and were able to order the features so as not to have to consider all possible feature sets; in practice, the features are not ordered, and a good feature set must be found from among all possible feature subsets. This involves the use of a feature-selection algorithm, which is part of the classification rule. Feature selection yields classifier constraint, not a reduction in the dimensionality of the feature space relative to design. For instance, if there are *d *features available for linear discriminant analysis (LDA), when used directly, then the classifier family consists of all hyperplanes in *d*-dimensional space. But, if a feature-selection algorithm reduces the number of variables to *m *<*d *prior to application of LDA, then the classifier family consists of all hyperplanes in *d*-dimensional space confined to *m*-dimensional subspaces. The dimensionality of the classification rule has not been reduced, but the new classification rule (feature selection plus LDA) is constrained.

A standard way of measuring classifier complexity is via the *VC *(*Vapnik-Chervonenkis*) *dimension *of a family of classifiers [3]. The VC dimension is defined in the Methods Section; here we note that it provides a measure of the degree to which a classifier can separate points, the greater the separation ability the higher the VC dimension and the lower the separation ability the lower the VC dimension. High VC-dimension classifiers have a greater ability to discriminate complex class interaction, at the cost of a greater potential to overfit, than do low VC-dimension classifiers. The VC dimension of a linear classifier with *d *features is *d *+ 1, whereas the VC dimension of a feed-forward neural network with one hidden layer, *d *features and *k *hidden nodes, exceeds *d*(*k *- 1) [4]. Depending on the number of nodes in the hidden layer, this can greatly exceed the VC dimension of a linear classifier.

The potential for overfitting is exhibited by a classical bound on the expected design error. For sample size *n*, the expected design cost of a classifier chosen from a family *G *via the empirical-error rule, which chooses the classifier in *G *that makes the least number of errors on the sample data, can be bounded via the VC dimension of *G*,

εG,n−εG≤VGlog⁡nnc0     (1)
 MathType@MTEF@5@5@+=feaafiart1ev1aaatCvAUfKttLearuWrP9MDH5MBPbIqV92AaeXatLxBI9gBaebbnrfifHhDYfgasaacH8akY=wiFfYdH8Gipec8Eeeu0xXdbba9frFj0=OqFfea0dXdd9vqai=hGuQ8kuc9pgc9s8qqaq=dirpe0xb9q8qiLsFr0=vr0=vr0dc8meaabaqaciaacaGaaeqabaqabeGadaaakeaaiiGacqWF1oqzdaWgaaWcbaGaem4raCKaeiilaWIaemOBa4gabeaakiabgkHiTiab=v7aLnaaBaaaleaacqWGhbWraeqaaOGaeyizIm6aaOqaaeaadaWcaaqaaiabdAfawnaaBaaaleaacqWGhbWraeqaaOGagiiBaWMaei4Ba8Maei4zaCMaemOBa4gabaGaemOBa4gaaaWcbaGaem4yam2aaSbaaWqaaiabicdaWaqabaaaaOGaaCzcaiaaxMaadaqadaqaaiabigdaXaGaayjkaiaawMcaaaaa@4743@

where *V*_*G *_is the VC dimension of *G *and *c*_0 _is a constant independent of *G *and *n *[4]. To make the bound small the sample size must greatly exceed the VC dimension. While providing a cautionary warning concerning high VC-dimension classifiers, the bound of Eq. 1 is not the end of the story when it comes to classifier design. First of all, the bound applies to all possible distributions, and therefore can be very loose. Second, and directly to the point of the present study, the design error depends on the classification rule, not only the family from which the classifier is chosen.

Given a very large sample, one might pose the rule of thumb that a more complex distribution requires a more complex classifier. The overfitting problem strikes at this rule of thumb when samples are insufficiently large. The rule is not reversed; rather, it breaks down. The VC dimension, or any other measure of classifier complexity, can only provide a loose guideline in the context of a warning against overfitting. The efficacy of a classifier design strategy depends not only on the complexity (VC dimension) of the classifier family, but also on the complexity of the feature-label distribution, or the difficulty of the classification problem. The latter can be rigorously approached by defining a measure of distributional complexity and calculating classifier performance as a function of distributional complexity [5]. In that case, when samples are small, as expected we observe that simple classifiers work best for low-complexity distributions, but we also often observe that, when a complex classifier performs well, the distribution is also of low complexity and a simple classifier could just as well do the job. This is why in microarray studies one should use high-complexity classifiers with caution.

The point of this paper is that one should not give up on complex classifiers, in particular, neural networks. As we will see in the current investigation of neural-network design, using an appropriate design strategy can yield good results in a small-sample setting, even for a high VC-dimension classifier. Specifically, neural networks can give competitive results for small samples, so long as they are properly trained. The aim of the present study is to examine over a range of models the degree to which variously trained neural networks can provide competitive results for the kinds of sample sizes used in many microarray studies, our emphasis being on training via noise injection.

### Noise injection

Sietsma and Dow [6] found that injecting noise into the sample data can lead to neural networks with improved performance, meaning that the designed neural networks can have smaller misclassification errors than those designed without noise injection. Since then there has been substantial research on two aspects of noise injection, theoretical proof and implementation. Holmsträom and Koistinen [7] showed that noise-injection-based design is asymptotically consistent as the sample size goes to infinity provided that the noise is chosen correctly. Moreover, rather than relying on heuristic choices, they developed rigorous methods to find the distribution of noise to be injected through Gaussian kernel density estimation. By using a Taylor expansion, Matsuoka [8] claimed that injecting noise to the neural network is equal to adding a regularization term to the error cost function. Following a similar but more rigorous approach, a neural network based on a regularized error function was introduced by Bishop [9] to avoid the randomness and increasing computation time brought by the injected random noise. The regularization term in the cost function penalizes a fast changing input-output relationship, and is believed to prevent the neural network from overfitting to the individual sample points. However, An [10] pointed out that the second derivative term of the cost function, which is omitted in Bishop's derivation, is a perturbation that can be either positive or negative, and can be ignored only when the network function fits well or is very smooth. Grandvalet and Cano [11] also found that the error perturbation cannot be omitted, and Grandvalet [12] found a bound for the perturbation as a function of the amount of noise injected. He therefore claimed that noise injection could be beneficial. In the mean time, Reed *et al*. [13] pointed out that noise injection has similar effects as weight decay, another common method used in neural network training in order to improve performance.

Recently, the study of noise injection has moved to improving the training scheme (classification rule). Proposed methods include applying the noise not to the input data but to the hidden nodes [14]; use of elliptic rather than spherical shaped Gaussian noise, and basing the noise distribution on the data [15]; noise designed according to the local data distribution [16]; and utilizing a regularized cost function in conjunction with noise injection to alleviate the effects caused by inappropriate variance of the injected noise [17].

**Figure 3:**
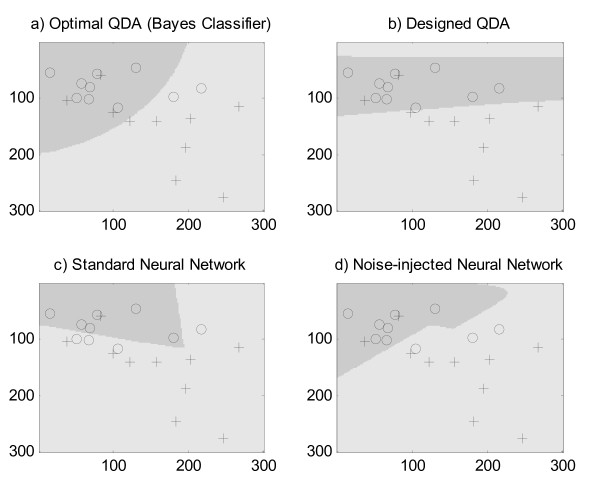
**A visual comparison of different classifiers**. The data model is a two-dimension nonlinear model. Sample size is 20, with 10 points from each class. The samples from class 0 and class 1 are denoted with round and cross markers, respectively. The shading denotes the decision region determined by the corresponding classifier, with darker shade for class 0 and lighter shade for class 1. The structure of the neural network is 2-4-2.

The effects of noise injection can be illustrated pictorially. Figure 3 corresponds to a 2-feature nonlinear model and shows a sample of size 20, with 10 points from each class. Parts a, b, c, and d of the figure show the optimal classifier, the quadratic discriminant analysis (QDA) classifier, the standard neural network (SNN), and the noise-injected neural network (NINN), respectively. The standard neural network overfits the training data by fitting its decision boundary into the small gaps between samples, either between two classes (the jutting right corner of the decision boundary), or among points of the same class (the left part of decision boundary, which cuts through four circle points). By filling up small gaps with noise points, noise injection has moved the decision boundary out of these local optimal states, smoothly fit it into the large gap between the two classes, so it is closer to the Bayes decision boundary, and hence enhances the classification performance.

## Results and discussion

In this study, we have conducted an extensive simulation-based comparative study of NINN design by taking advantage of contemporary high-performance computing, a 512-node Beowulf cluster, which was not available when many neural-network training procedures were proposed. We consider a number of different feature-label models across various small sample sizes using varying amounts of noise injection. Besides comparing NINN design to classical SNN design, the paper compares it to a number of other classification rules: LDA, QDA, the strong feature classifier (SFC), the Gaussian kernel (GK) classifier, and the 3-nearest-neighbor (3NN) classifier. Since our applications concern microarray data for expression-based classification for diagnosis and prognosis, we also consider NINN design as it relates to a study of survivability of breast cancer patients. The models and patient data are described in the Methods Section, as are the classification rules used in the study.

Corresponding to each experimental case determined by the model, the degree of correlation, and the number of features, there is a graph of the errors of the classifiers for the sample sizes considered. The complete set of these graphs appear in the additional file 1, with some of them also appearing in the paper to support the discussion. In these comparison graphs, the noise NINN results correspond to largest amount of noise injection available (check Method section for details). The amount of noise injected is important. For each situation there is a graph showing the errors for different amounts of noise for each sample size considered, again the full set being given in the additional file 1. A large amount of noise may be required to achieve good noise-injected design and this entails a high computational cost, which can be prohibitive when considering a large number of feature sets. In the case of linear classification, avoidance of this computational burden motivated the introduction of SFC, which produces the effect of noise injection but determines the classifier analytically without the computational cost of introducing random noise. Let us proceed to consider the different models.

### Linear model

**Figure 4:**
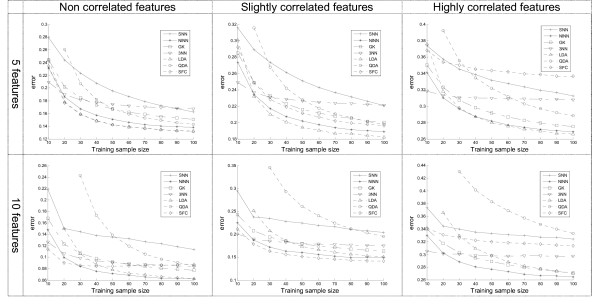
Comparison of various classifiers for linear model.

Simulation results for the linear model are shown in Fig. 4. For the linear model, LDA is optimal for the feature-label distribution, with its sample-based performance depending on sample size. In the case of 5 uncorrelated features, except for the impractical case of *n *= 10, LDA and SFC perform equivalently, with QDA in the processing of catching up as the sample size increases, as expected. Among the neural nets, NINN performs best and its performance is very close to that of LDA. As is the case throughout the experiments, SNN does poorly. For 10 uncorrelated features, the performance of all the classifiers improves, but now we see the advantage of SFC for very small samples. It outperforms LDA for *n *< 40, after which it stabilizes, whereas LDA continues to improve. Most interestingly relative to the current study, for *n *≥ 20, NINN outperforms LDA. For both 5 and 10 features, GK is not far behind NINN and 3NN is not competitive with NINN except for very small sample sizes.

For slightly correlated features, the situation is mostly analogous to the uncorrelated case, except that overall performance is worse, as expected. The main difference is the performance decline of SFC relative to LDA and NINN, although SFC still performs better than QDA, and SNN. Matters are quite different for 10 features. SFC outperforms LDA and NINN for all sample sizes, SFC and NINN significantly outperforming LDA for smaller sample sizes. The key point is that LDA is only performing slightly better with 10 features than with 5. As seen in previous cases, 3NN performs best for very small samples.

When we go to highly correlated features, there is severe performance degradation. For 5 features, NINN bests LDA for *n *≤ 20, and their performance is essentially the same for *n *≥ 30. GK is not far behind. The more interesting observations occur with 10 features. Except for *n *≤ 20, when 3NN is better, NINN provides the best performance, and is much better than LDA for the smaller sample sizes. NINN is also better than GK, although the latter outperforms LDA for *n *≤ 70. The problem with LDA is that it has suffered the peaking phenomenon: it performs worse with 10 features than with 5. Early peaking for LDA with highly correlated features has been previously observed [2]. Note that in this highly correlated case, as with the previous cases, 3NN performs well for very small samples, but does not improve much thereafter as the sample size increases.

**Figure 5:**
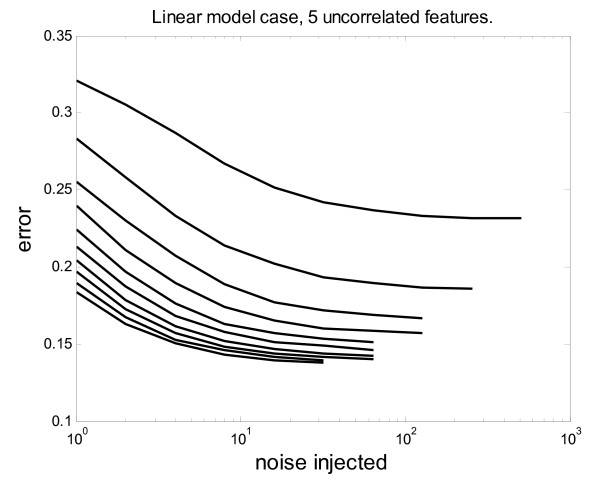
**Effects of different amounts of noise for uncorrelated linear model**. Classification error is plotted as a function of the amount of noise injection. Each line corresponds to a fixed training sample size, from 10 to 100 for every 10 sample points, the highest line corresponding to 10 sample points and the lowest corresponding to 100 sample points.

The effect of different amounts of noise is shown in Fig. 5 for uncorrelated features. Classification error is plotted as a function of the amount of noise injection. Each line corresponds to a fixed training sample size, from 10 to 100 for every 10 sample points, the highest line corresponding to 10 sample points and the lowest corresponding to 100 sample points. This graph is typical in that noise injection is more beneficial for smaller samples and there is diminishing return for additional noise. Indeed, too much noise can mask the original data to the extent that it ceases to be beneficial. Throughout the experiments we have seen that 10-fold noise injection is quite beneficial and typically does not cause loss of performance. Note that the graph in Fig. 5 has substantially more noise injected for smaller samples. This is on account of two reasons: first, more added noise provides greater benefit for smaller samples; second, the computational burden increase in accordance to the sample size. Owing to the typical behaviour illustrated in Fig. 5, for all other models we defer to the additional file 1 for graphs showing the effects of different amounts of noise injection.

### Low-curvature nonlinear model

**Figure 6:**
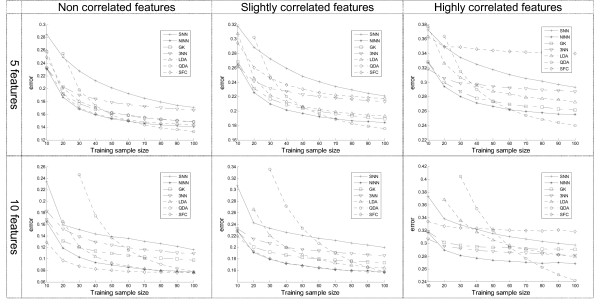
Comparison of various classifiers for low-curvature nonlinear model.

Simulation results for the low-curvature nonlinear model are shown in Fig. 6. As expected, although QDA is optimal for the feature-label distribution, LDA outperforms QDA for smaller sample sizes (*n *≤ 60) owing to its lower complexity. From the perspective here, the key point is that NINN performs best among all classifiers for *n *≤ 60 and second to QDA (and only slightly worse) for *n *≥ 70. Note that GK and SFC are not far behind NINN. An interesting phenomenon occurs with 10 features: SFC performs best for *n *≤ 90. Along with this, LDA significantly outperforms QDA. As for NINN, it is close behind SFC, especially for *n *≥ 40.

For slightly correlated features, there are some large differences in the performance comparisons. In this case, with 5 features SFC performs relatively very poorly. QDA overtakes LDA earlier and then does much better as the sample size increases. However, once again NINN does well, having the best performance for 20 ≤ *n *≤ 60, and only being bested by QDA for *n *≥ 70. Note also that GK performs close to NINN for all *n*. Similar statements hold for 10 features, an exception being that QDA never overtakes NINN.

With highly correlated features, peaking plays a critical role. It is particularly severe for LDA and QDA, with error rates higher for 10 features than for 5. This is in agreement with the results of previous study as shown in Fig. 2[2]. It even occurs for NINN and GK. Otherwise, there are a lot of similarities to the slightly correlated case, with QDA overtaking LDA and, as in the 5-feature slightly correlated case, NINN outperforming QDA for the smaller sample sizes, this time in both the 5- and 10-feature cases. An interesting point regarding neural networks is that, for 5 features, GK is not far behind NINN, as we have witnessed before.

### High-curvature nonlinear model

**Figure 7:**
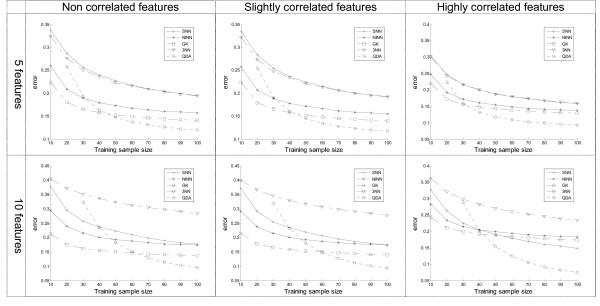
Comparison of various classifiers for high-curvature nonlinear model.

Simulation results for the high-curvature nonlinear model are shown in Fig. 7. It is instructive to compare the results for the low-curvature and high-curvature models. Focusing first on 5 features, in the uncorrelated model we are struck by the much poorer relative performance of NINN for the high-curvature model in comparison to the low-curvature model. Whereas in the low-curvature nonlinear model, NINN is substantially better than SNN, and slightly better than GK, in the high-curvature nonlinear model, NINN is bested by GK, although it remains significantly better than SNN. Its relation to QDA is also much different in the high-curvature model, where QDA is superior to it for *n *≥ 50, and much better than it for *n *≥ 60, whereas in the low-curvature model, QDA does not outperform NINN until *n *≥ 70, and then not very much. For 10 features in the uncorrelated case, NINN again outperforms SNN across all sample sizes, the gap closing at sample size 100. As in the 5-feature case, NINN is beat by GK. Whereas QDA never surpasses NINN in the low-curvature model, it surpasses NINN in the high-curvature model for *n *≥ 50. Analogous considerations apply to the different relative performance of NINN compared to GK and QDA in the other five high-curvature models.

Relative to overfitting the sample data, the key difference between the low-curvature and high-curvature nonlinear models is the increased curvature in the high-curvature model. Noise injection has the effect of smoothing the decision boundary and this smoothing has greater benefit when the decision boundary is less curved. As another effect of high curvature, note the strikingly poor performance of 3NN.

### Equal-mean model

In this model, QDA is optimal for the feature-label distribution with the decision boundary being a hypersphere. The main point to be made here is that the comparisons are similar to the high-curvature nonlinear model, the key factor being the high curvature of the decision boundary in the QDA-optimal model. Another noteworthy observation is that the performance of NINN does not always monotonically improve along with the amount of noise injected. In some certain cases, the classification error can first decrease, then increase after the noise injection surpasses a certain amount. This phenomenon is most prominent in the case of 10 highly correlated features.

### XOR model

The results for the XOR model are fairly consistent and clear across all six cases, 5 and 10 features, and uncorrelated, slightly correlated, and highly correlated models. NINN, GK, and 3NN have very close performances and they perform significantly better than SNN. If anything can be said concerning the relationship between NINN, GK, and 3NN, it is that 3NN is insignificantly slightly better than NINN and GK for small sample sizes, with the situation reversing for larger sample sizes.

### Bimodal model

**Figure 8:**
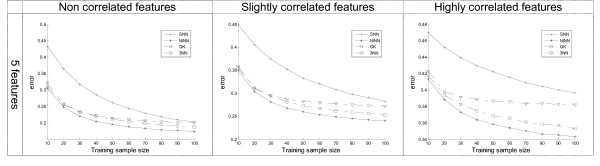
Comparison of various classifiers for bimodal model.

Simulation results for the 5-feature case for the bimodal model are shown in Fig. 8. The results for the bimodal model show similarity to those for the XOR model in that, as a group, NINN, GK, and 3NN tend to outperform SNN for the smaller sample sizes, but with the intra-group performances more spread and the inter-group performance differences tending to dissipate as the sample size increases. Among the classifiers NINN, GK, and 3NN, for 5 features, NINN is better than GK, which is better than 3NN, with the differences becoming larger for increasing correlation. For 10 features, NINN remains the best but 3NN outperforms GK.

### Patient data

**Figure 9:**
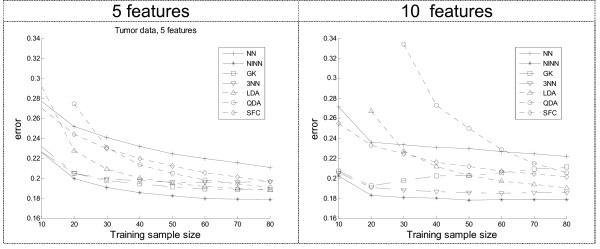
Comparison of various classifiers for patient data.

Simulation results for the patient data are shown in Fig. 9. Basically, our simulation on patient data shows that NINN achieves the best performance with 3NN, GK and LDA not too far behind.

When we observe the 5-feature results for the patient data, we see a striking similarity with those for slightly correlated features in the nonlinear model. Ignoring the fact that QDA provides the best performance for larger samples in the nonlinear model, for which it is optimal relative to the feature-label distribution, in both cases NINN performs best across the full range of sample sizes, and even more so with the patient data. In both cases, LDA and GK are similar and trail NINN. 3NN is a little better in the real patient data while SFC is a little worse. Note that while we have compared the patient-data results to those of the slightly correlated nonlinear model, similar correspondences exist between the patient data and the highly correlated nonlinear model, which is only reasonable since, as pointed out previously, there are many similarities between the slightly and highly correlated nonlinear models.

The salient point regarding using 10 features for the patient data is peaking, as it is in the highly correlated nonlinear model. For instance, for *n *= 40, LDA performs worse with 10 features than with 5 features and QDA performs much worse with 10 features than with 5. NN shows no improvement with 10 features compared with 5 features. NINN shows a slight improvement with 10 features, indicating later peaking. Although NINN pretty much flattens out when *n *> 20, it again has the best performance across all sample sizes. The most prominent differences between the real patient data and highly correlated nonlinear model are QDA and GK. Poor performance owing to peaking is particularly evident with GK.

## Conclusion

Although neural networks have high VC dimension and can therefore suffer from overfitting the sample data, their performance is highly dependent on the training procedure (classification rule) employed. This paper has demonstrated that in many instances noise-injected neural network design is superior to classical neural-network design and to the other tested methods, and in almost all cases it does not perform substantially worse than the best of the other methods. This conclusion has importance for the design of classifiers for diagnosis and prognosis based on gene-expression data because sample sizes are often limited and, unless the class conditional distributions are easily discriminated, say by a linear classifier, a higher-complexity classifier must be employed.

## Methods

Simulations have been conducted using both synthetic data and real patient data. For synthetic data, we have considered six different distribution models for data generation.

▪ **Linear model**: The class-conditional distributions are Gaussian, with *S*_0_~ *N*(**μ**_0_, **Σ**_0_) and *S*_1_~ *N*(**μ**_1_, **Σ**_1_), where **μ**_0 _= (0, 0,..., 0), **μ**_1 _= (1, 1,..., 1) and **Σ**_0 _= **Σ**_1 _= **Σ**. The corresponding Bayes classifier is linear and the Bayes decision boundary is a hyperplane and can be found via linear discriminant analysis (LDA).

▪ **Low-curvature nonlinear model**: The class-conditional distributions are Gaussian with unequal variance matrices: **μ**_0 _= (0, 0,..., 0), **μ**_1 _= (1, 1,..., 1), and 2**Σ**_0 _= **Σ**_1 _= 2**Σ**. The Bayes classifier is nonlinear, the Bayes decision boundary is quadratic and can be found via quadratic discriminant analysis (QDA), and the boundary possesses low curvature in comparison to the high-curvature nonlinear model to be described next.

▪ **High-curvature nonlinear model**: This model and the following equal-mean model have been used in [7] and [18]. The class-conditional distributions are Gaussians: **μ**_0 _= (0, 0,..., 0), **μ**_1 _= (2.32, 0,..., 0) and 4**Σ**_0 _= **Σ**_1 _= 4**Σ**. The Bayes decision boundary is quadratic, it is found via QDA, and it possesses higher curvature than the decision boundary for the low-curvature nonlinear model.

▪ **Equal-mean model**: The class-conditional distributions are Gaussians. Both classes share the same mean vector, **μ**_0 _= **μ**_1 _= (0, 0,..., 0), with 4**Σ**_0 _= **Σ**_1 _= 4**Σ**. The Bayes decision boundary is a hypersphere determined by QDA, the covariance structure is the same as in the high-curvature nonlinear model, the decision boundary has high curvature in comparison to the low-curvature nonlinear model, and this model is more difficult than the high-curvature nonlinear model.

▪ **XOR model**: The class-conditional distributions of both classes are mixture of two equi-probable Gaussians. The covariance matrices of the classes are identical, i.e., **Σ**_00 _= **Σ**_01 _= **Σ**_10 _= **Σ**_11 _= **Σ**. The mean vectors of class *S*_0 _are **μ**_00 _= (1, -1, 1,...), **μ**_01_= (-1, 1, -1,...). The mean vectors of class *S*_1 _are **μ**_10 _= (1, 1, 1,...), **μ**_11 _= (-1, -1, -1,...). The Bayes decision boundaries are two perpendicular hyperplanes.

▪ **Bimodal model**: The class-conditional distribution of class *S*_0 _is Gaussian, centered at **μ**_0 _= (0, 0,...,0), and the class-conditional distribution of class *S*_1 _is a mixture of two equi-probable Gaussians, centered at **μ**_10 _= (1, 1,..., 1) and **μ**_11_= (-1, -1,..., -1). The covariance matrices of the classes are identical, i.e., **Σ**_0 _= **Σ**_10 _= **Σ**_11 _= **Σ**. The Bayes decision boundaries are two parallel hyperplanes.

Throughout the simulation, we assume that the two classes have equal prior probability.

We assume the covariance matrix **Σ **has a spherical structure such that every two features possess the same correlation, namely,

∑=σ2[1ρ⋯⋯⋯ρρ1⋮⋮⋱⋮⋮⋱⋮⋮1ρρ⋯⋯⋯ρ1],     (2)
 MathType@MTEF@5@5@+=feaafiart1ev1aaatCvAUfKttLearuWrP9MDH5MBPbIqV92AaeXatLxBI9gBaebbnrfifHhDYfgasaacH8akY=wiFfYdH8Gipec8Eeeu0xXdbba9frFj0=OqFfea0dXdd9vqai=hGuQ8kuc9pgc9s8qqaq=dirpe0xb9q8qiLsFr0=vr0=vr0dc8meaabaqaciaacaGaaeqabaqabeGadaaakeaacqGHris5cqGH9aqpiiGacqWFdpWCdaahaaWcbeqaaiabikdaYaaakmaadmaabaqbaeqabyGbaaaaaeaacqaIXaqmaeaacqWFbpGCaeaacqWIVlctaeaacqWIVlctaeaacqWIVlctaeaacqWFbpGCaeaacqWFbpGCaeaacqaIXaqmaeaaaeaaaeaaaeaacqWIUlstaeaacqWIUlstaeaaaeaacqWIXlYtaeaaaeaaaeaacqWIUlstaeaacqWIUlstaeaaaeaaaeaacqWIXlYtaeaaaeaacqWIUlstaeaacqWIUlstaeaaaeaaaeaaaeaacqaIXaqmaeaacqWFbpGCaeaacqWFbpGCaeaacqWIVlctaeaacqWIVlctaeaacqWIVlctaeaacqWFbpGCaeaacqaIXaqmaaaacaGLBbGaayzxaaGaeiilaWIaaCzcaiaaxMaadaqadaqaaiabikdaYaGaayjkaiaawMcaaaaa@6242@

where *σ *is the standard deviation of each feature, and *ρ *the correlation between features. If *ρ *= 0, then all features are uncorrelated. As *ρ *increases, the correlation among features increases. We consider three representative covariance matrices by setting *ρ *equal to 0, 0.125 and 0.5, and referring to these as *uncorrelated features*, *slightly correlated features *and *highly correlated features*, respectively. Note that in [7] and [18], no correlation among features is considered when they discuss the nonlinear and equal-mean models. As for the feature size, two feature sizes, 5 and 10, are tested in our simulation. By considering all the possible combinations of the distribution model, feature size and covariance matrix, there are altogether 36 different situations.

The patient data come from a microarray cancer-classification study that analyzes a large number of microarrays prepared with RNA from breast tumor samples of 295 patients [19]. Using a previously established 70-gene prognosis profile [20], a prognosis signature based on gene-expression that correlates well with patient survival data and other existing clinical measures is proposed in [19]. Of the 295 microarrays, 115 belong to the 'good-prognosis' class and the other 180 belong to the 'poor-prognosis' class.

Seven classifiers are considered in the study: standard neural network (SNN) [21], neural network designed with noise injection (NINN) [7], the Gaussian kernel (GK) classifier [4], LDA [21], QDA [21], the strong-feature classifier (SFC) [22], and the 3-nearest-neighbor (3NN) classifier [4]. We include LDA and QDA because with full distributional knowledge they are optimal in the linear and nonlinear models, respectively, with QDA also optimal in the equal-mean model. We include SFC because, by replacing each data point with a spherical Gaussian distribution and then finding the classifier via a Wiener-filter methodology, it provides an analytic form of noise injection that is much more computationally efficient than the addition of random noise. Owing to the kinds of problems they are meant to address, we apply LDA and SFC only in the linear and nonlinear models, and we apply QDA in the linear, nonlinear, and equal-mean models. We include 3NN for comparison purposes because it has been used extensively in expression-based analysis and, as we will see, performs relatively well on very small samples.

We use feed-forward layered networks with one hidden layer. For 5 and 10 features, the network structures are 5-8-2 and 10-15-2, respectively, meaning there are 8 and 15 nodes in the hidden layer, respectively. Since the classifiers are binary, there are two units in the output layer. Normalization of input data and initialization of the parameters inside the network are done according to [21]. Error back-propagation is used to calculate the derivatives of the cost function, which is minimized by using the Levenberg-Marquardt method [23]. The source code of the authors' neural network implementation is available on request.

For the GK classifier, assume there are *n *training sample points, (***x***_***i***_, *y*_*i*_), i = 1, 2,..., *n*, with sample point ***x***_***i ***_a *d*-dimensional vector, and *y*_*i *_its label. Then for a testing point ***x***, the posterior probability of its label *y *being *k *is estimated by

P(y=k)=∑i=1nI(yi=k)⋅(2π⋅hi2)−1/2dexp⁡(−‖x−xi‖2/(2hi2)),     (3)
 MathType@MTEF@5@5@+=feaafiart1ev1aaatCvAUfKttLearuWrP9MDH5MBPbIqV92AaeXatLxBI9gBamXvP5wqSXMqHnxAJn0BKvguHDwzZbqegyvzYrwyUfgarqqtubsr4rNCHbGeaGqiA8vkIkVAFgIELiFeLkFeLk=iY=Hhbbf9v8qqaqFr0xc9pk0xbba9q8WqFfeaY=biLkVcLq=JHqVepeea0=as0db9vqpepesP0xe9Fve9Fve9GapdbaqaaeGacaGaaiaabeqaamqadiabaaGcbaGaemiuaaLaeiikaGIaemyEaKNaeyypa0Jaem4AaSMaeiykaKIaeyypa0ZaaabCaeaacqWGjbqsdaqadaqaaiabdMha5naaBaaaleaacqWGPbqAaeqaaOGaeyypa0Jaem4AaSgacaGLOaGaayzkaaaaleaacqWGPbqAcqGH9aqpcqaIXaqmaeaacqWGUbGBa0GaeyyeIuoakiabgwSixpaabmaabaGaeGOmaidcciGae8hWdaNaeyyXICTaemiAaG2aa0baaSqaaiabdMgaPbqaaiabikdaYaaaaOGaayjkaiaawMcaamaaCaaaleqabaGaeyOeI0IaeGymaeJaei4la8IaeGOmaiJaemizaqgaaOGagiyzauMaeiiEaGNaeiiCaa3aaeWaaeaacqGHsisldaqbdaqaaiabdIha4jabgkHiTiabdIha4naaBaaaleaacqWGPbqAaeqaaaGccaGLjWUaayPcSdWaaWbaaSqabeaacqaIYaGmaaGccqGGVaWldaqadaqaaiabikdaYiabdIgaOnaaDaaaleaacqWGPbqAaeaacqaIYaGmaaaakiaawIcacaGLPaaaaiaawIcacaGLPaaacqGGSaalcaWLjaGaaCzcamaabmaabaGaeG4mamdacaGLOaGaayzkaaaaaa@81F9@

where *I*() is the identity function, and *h*_*i *_is the smoothing factor which is chosen in the same way as in the noise-injection procedure to be shortly described. The GK classifier will pick the *k *with the larger posterior probability as the predicted label.

For NINN design we use the method in [7]. Again let there be *n *sample points, (***x***_**1**_, *y*_1_), (***x***_**2**_, *y*_2_), ..., (***x***_***n***_, *y*_*n*_). The amount of noise injected is measured by the ratio between the noise-injected sample size and the original sample size. In our simulation, we ensure that the same number of noise points is generated for each training sample point. For instance, if the noise injection amount is *k*, then our sampling procedure is performed in the following manner:

1) Pick ***x***_***i ***_from the training sample;

2) Draw a point ***z ***from standard normal distribution, ***z ***~ *N*(**0**, **1**);

Generate a noise point by ***x ***= ***x***_***i ***_+ *h*_*i*_***z***, where *h*_*i *_is the smoothing factor for point *x*_*i*_, and is given by [7]

*h*_*i *_= (8^*d*/4-1^*d*^*d*/2^Γ(*d*/2))^2/(*d*+4) ^*n*^-1/(*d*+4) ^(*σ*_0_*I*(*y*_*i *_= 0) + *σ*_1_*I*(*y*_*i *_= 1)),     (4)

where *I*() is identity function, and *σ*_0 _and *σ*_1 _are the estimated standard deviations of class 0 and 1, respectively;

3) Repeat steps 2 and 3 *k* times to generate *k *noise points around ***x***_***i***_;

4) Repeat steps 1 through 4 for *i *= 1, 2, ..., *n *to generate *kn *noise points.

To test the effects of different amounts of noise injection, for each sample size *n*, we allow *k *= 2^*b*^, *b *= 0, 1,..., *B*, where *B *is the largest integer that *kn = *2^*B*^*n *≤ 5120, in the simulation. We set 5120 as the maximum sample size after noise injection to avoid too much computation, owing to the slow convergence in the training of the neural network. Note that the original sample points are not used for the final training of the network, so for noise-injection amount 2^0 ^= 1, the result is simply a perturbation of the original data. When comparing with other classifiers, the results with the largest amount of noise 2^*B *^are used.

For synthetic data, the simulation is done by independently applying each classifier to different situations. For each situation, the simulation generates *n *training points (*n*/2 points for each class) according to the distribution model, feature size, and covariance matrix of the corresponding situation. The trained classifier is applied to 200 independently generated test points from the identical distribution. This procedure is repeated 10,000 times for all classifiers, and for NINN, for all possible noise injection amounts. The training sample size varies from 10 to 100, with increase by steps of 10. The entire simulation is repeated for different training sample sizes, feature sizes, and situations.

For patient data, we apply all seven classifiers to the patient data and estimate the error by using a hold-out method. For a sample size of *n*, *n *sample points are drawn without replacement from the 295 data points. Out of the 70 genes, *d *are selected based on the *n *training points in the following manner: for each gene, we calculate the difference of the mean expression values between the two classes, normalize this value by the sum of the corresponding standard deviations, and then select the genes with largest differences [24]. To make a straightforward comparison between the results of the patient data and synthetic data, here we choose the same feature sizes as we do in the synthetic data simulation, i.e., *d *= 5 and 10. The classifier trained on the *n *points is tested on the 295 – *n *points not drawn. This procedure is repeated 5000 times and error rates are averaged to obtain an estimate of the sample-based classification error. As discussed for this hold-out procedure using the same data set in [25], since in the hold-out experiment the observations are not fully independent, we limit *n *to under 80 to reduce the impact of observation correlation.

### VC dimension

To define the VC dimension of a classifier family *C*, for each classifier ψ ∈ *C *consider the set, {(**x**, *y*): ψ(**x**) ≠ *y*}, of all points in the feature-label space ℜ^*d *^× {0, 1) for which the value of the classifier at point **x **does not equal the value of the label at **x**. Let A
 MathType@MTEF@5@5@+=feaafiart1ev1aaatCvAUfKttLearuWrP9MDH5MBPbIqV92AaeXatLxBI9gBamrtHrhAL1wy0L2yHvtyaeHbnfgDOvwBHrxAJfwnaebbnrfifHhDYfgasaacH8akY=wiFfYdH8Gipec8Eeeu0xXdbba9frFj0=OqFfea0dXdd9vqai=hGuQ8kuc9pgc9s8qqaq=dirpe0xb9q8qiLsFr0=vr0=vr0dc8meaabaqaciaacaGaaeqabaWaaeGaeaaakeaaimaacqWFaeFqaaa@3821@_*C *_be the collection of these sets for all ψ ∈ *C*. If {**z**_1_, **z**_2_,..., **z**_*m*_} is a set of *m *points in ℜ^*d*^, let *N*_*C *_(**z**_1_, **z**_2_,..., **z**_*n*_) be the number of distinct subsets of {**z**_1_, **z**_2_,..., **z**_*m*_} created by intersection with sets in A
 MathType@MTEF@5@5@+=feaafiart1ev1aaatCvAUfKttLearuWrP9MDH5MBPbIqV92AaeXatLxBI9gBamrtHrhAL1wy0L2yHvtyaeHbnfgDOvwBHrxAJfwnaebbnrfifHhDYfgasaacH8akY=wiFfYdH8Gipec8Eeeu0xXdbba9frFj0=OqFfea0dXdd9vqai=hGuQ8kuc9pgc9s8qqaq=dirpe0xb9q8qiLsFr0=vr0=vr0dc8meaabaqaciaacaGaaeqabaWaaeGaeaaakeaaimaacqWFaeFqaaa@3821@_*C*_. The *m*^th ^*shatter coefficient *of *C*, denoted by ξ(A
 MathType@MTEF@5@5@+=feaafiart1ev1aaatCvAUfKttLearuWrP9MDH5MBPbIqV92AaeXatLxBI9gBamrtHrhAL1wy0L2yHvtyaeHbnfgDOvwBHrxAJfwnaebbnrfifHhDYfgasaacH8akY=wiFfYdH8Gipec8Eeeu0xXdbba9frFj0=OqFfea0dXdd9vqai=hGuQ8kuc9pgc9s8qqaq=dirpe0xb9q8qiLsFr0=vr0=vr0dc8meaabaqaciaacaGaaeqabaWaaeGaeaaakeaaimaacqWFaeFqaaa@3821@, *m*), is the maximum value of *N*_*C *_(**z**_1_, **z**_2_,..., **z**_*n*_) over all point sets {**z**_1_, **z**_2_,..., **z**_*m*_}. If ξ (A
 MathType@MTEF@5@5@+=feaafiart1ev1aaatCvAUfKttLearuWrP9MDH5MBPbIqV92AaeXatLxBI9gBamrtHrhAL1wy0L2yHvtyaeHbnfgDOvwBHrxAJfwnaebbnrfifHhDYfgasaacH8akY=wiFfYdH8Gipec8Eeeu0xXdbba9frFj0=OqFfea0dXdd9vqai=hGuQ8kuc9pgc9s8qqaq=dirpe0xb9q8qiLsFr0=vr0=vr0dc8meaabaqaciaacaGaaeqabaWaaeGaeaaakeaaimaacqWFaeFqaaa@3821@, *m*) = 2^*m*^, then *C *is said to *shatter *{**z**_1_, **z**_2_,..., **z**_*m*_}. This means there is at least one set of *m *points for which all subsets of the set can be constructed by intersection with sets in A
 MathType@MTEF@5@5@+=feaafiart1ev1aaatCvAUfKttLearuWrP9MDH5MBPbIqV92AaeXatLxBI9gBamrtHrhAL1wy0L2yHvtyaeHbnfgDOvwBHrxAJfwnaebbnrfifHhDYfgasaacH8akY=wiFfYdH8Gipec8Eeeu0xXdbba9frFj0=OqFfea0dXdd9vqai=hGuQ8kuc9pgc9s8qqaq=dirpe0xb9q8qiLsFr0=vr0=vr0dc8meaabaqaciaacaGaaeqabaWaaeGaeaaakeaaimaacqWFaeFqaaa@3821@_*C*_. The shatter coefficient of *C *measures the extent to which the sets in A
 MathType@MTEF@5@5@+=feaafiart1ev1aaatCvAUfKttLearuWrP9MDH5MBPbIqV92AaeXatLxBI9gBamrtHrhAL1wy0L2yHvtyaeHbnfgDOvwBHrxAJfwnaebbnrfifHhDYfgasaacH8akY=wiFfYdH8Gipec8Eeeu0xXdbba9frFj0=OqFfea0dXdd9vqai=hGuQ8kuc9pgc9s8qqaq=dirpe0xb9q8qiLsFr0=vr0=vr0dc8meaabaqaciaacaGaaeqabaWaaeGaeaaakeaaimaacqWFaeFqaaa@3821@_*C *_can separate points for various point-set sizes. The largest integer *m *for which ξ (A
 MathType@MTEF@5@5@+=feaafiart1ev1aaatCvAUfKttLearuWrP9MDH5MBPbIqV92AaeXatLxBI9gBamrtHrhAL1wy0L2yHvtyaeHbnfgDOvwBHrxAJfwnaebbnrfifHhDYfgasaacH8akY=wiFfYdH8Gipec8Eeeu0xXdbba9frFj0=OqFfea0dXdd9vqai=hGuQ8kuc9pgc9s8qqaq=dirpe0xb9q8qiLsFr0=vr0=vr0dc8meaabaqaciaacaGaaeqabaWaaeGaeaaakeaaimaacqWFaeFqaaa@3821@, *m*) = 2^*m *^is called the *Vapnik-Chervonenkis *(*VC*) *dimension *of *C*. If ξ (A
 MathType@MTEF@5@5@+=feaafiart1ev1aaatCvAUfKttLearuWrP9MDH5MBPbIqV92AaeXatLxBI9gBamrtHrhAL1wy0L2yHvtyaeHbnfgDOvwBHrxAJfwnaebbnrfifHhDYfgasaacH8akY=wiFfYdH8Gipec8Eeeu0xXdbba9frFj0=OqFfea0dXdd9vqai=hGuQ8kuc9pgc9s8qqaq=dirpe0xb9q8qiLsFr0=vr0=vr0dc8meaabaqaciaacaGaaeqabaWaaeGaeaaakeaaimaacqWFaeFqaaa@3821@, *m*) = 2^*m *^for all *m*, then the VC dimension is ∞. For a comprehensive discussion of the VC dimension and its implications, see [4].

## Authors' contributions

JH developed the models and classification rules, collaborated in the analysis, and helped draft the manuscript. JL developed efficient implementations of the various rules and experiments on the Beowulf cluster so that the massive simulation could be completed. ZX participated in the analysis and interpretation of the results. ED conceived the study, participated in the analysis and interpretation of the results, and helped draft the manuscript.

## Supplementary Material

Additional File 1 **Supplementary_Simulation_Results**. Complete simulation results are provided in this file. Click here for file

## References

[B1] Dougherty ER (2001). Small sample issues for microarray-based classification. Comparative and Functional Genomics.

[B2] Hua J, Xiong Z, Lowey J, Suh E, Dougherty ER (2005). Optimal number of features as a function of sample size for various classification rules. Bioinformatics.

[B3] Vapnik V, Chervonenkis A (1971). On the uniform convergence of relative frequencies of events to their probabilities. Theor Prob Appl.

[B4] Devroye L, Györfi L, Lugosi G (1996). A Probabilistic Theory of Pattern Recognition.

[B5] Attoor SN, Dougherty ER (2004). Classifier performance as a function of distributional complexity. Pattern Recognition.

[B6] Sietsma J, Dow RJF (1991). Creating artificial neural networks that generalize. Neural Networks.

[B7] Holmsträom L, Koistinen P (1992). Using additive noise in back-propagation training. IEEE Trans Neural Networks.

[B8] Matsuoka K (1992). Noise injection into inputs in back-propagation learning. IEEE Trans Syst Man and Cybern.

[B9] Bishop CM (1995). Training with noise is equivalent to Tikhonov regularization. Neural Computation.

[B10] An G (1996). The effects of adding noise during backpropagation traning on a generalization performance. Neural Computation.

[B11] Grandvalet Y, Canu S (1995). Comments on "Noise injection into inputs in back-propagation learning". IEEE Trans Syst Man and Cybern.

[B12] Grandvalet Y, Canu S, Boucheron S (1997). Noise injection: theoretical prospects. Neural Computation.

[B13] Reed R, Marks RJ, Oh S (1995). Similarities of error regularization, sigmoid gain scaling, target smoothing, and training with jitter. IEEE Trans Neural Networks.

[B14] Hammadi NC, Ito H (1998). Improving the performance of feedforward neural networks by noise injection into hidden neurons. J Intell Robot Syst.

[B15] Grandvalet Y (2000). Anisotropic noise injection for input variables relevance determination. IEEE Trans Neural Networks.

[B16] Skurichina M, Raudys S, Duin RPW (2002). K-Nearest neighbors directed noise injection in multilayer perceptron training. IEEE Trans Neural Networks.

[B17] Seghouane A, Moudden Y, Fleury G (2004). Regularizing the effect of input noise injection in feedforward neural networks training. Neural Comput & Applic.

[B18] Kohonen T, Barna G, Chrisley R (2001). Statistical pattern recognition with neural networks: Bechmarking studies. San Diego:Proc IEEE Int Conf Neural Networks.

[B19] van de Vijver MJ, He YD, van't Veer LJ, Dai H, Hart AAM, Voskuil DW, Schreiber GJ, Peterse JL, Roberts C, Marton MJ, Parrish M, Atsma D, Witteveen A, Delahaye L, van der Velde T, Bartelink H, Rodenhuis S, Rutgers ET, Friend SH, Bernards R (2002). A gene-expression signature as a predictor of survival in breast cancer. New Eng J Med.

[B20] van't Veer LJ, Dai H, van de Vijver MJ, He YD, Hart AAM, Mao M, Peterse HL, van der Kooy K, Marton MJ, Witteveen AT, Schreiber GJ, Kerkhoven RM, Roberts C, Linsley PS, Bernards R, Friend SH (2002). Gene expression profiling predicts clinical outcome of breast cancer. Nature.

[B21] Duda R, Hart P, Stork DG (2001). Pattern Classification.

[B22] Kim S, Dougherty ER, Barrera J, Chen Y, Bittner ML, Trent JM (2002). Strong feature sets from small samples. Journal of Computational Biology.

[B23] Press WH, Teukolsky SA, Vetterling WT, Flannery BP (2002). Numerical Recipes in C.

[B24] Golub TR, Slonim DK, Tamayo P, Huard C, Gaasenbeek M, Mesirov JP, Coller H, Loh ML, Downing JR, Caligiuri MA, Bloomfield CD, Lander ES (1999). Molecular classification of cancer: Class discovery and class prediction by gene expression monitoring. Science.

[B25] Braga-Neto U, Dougherty ER (2004). Is cross-validation valid for small-sample microarray classification?. Bioinformatics.

