# Supplementary material for: Noise-injected neural networks show promise for use on small-sample expression data
Source: BMC Bioinformatics. 2006 May 31;7:274. doi: 10.1186/1471-2105-7-274 (PMC1524820; doi:10.1186/1471-2105-7-274)
Supplement: Additional File 1 — Supplementary_Simulation_Results. Complete simulation results are provided in this file. [file 1471-2105-7-274-S1.pdf]

Supplementary Simulation Results For Noise-Injected Neural Networks Show Promise for use on Expression Data

Jianping Hua[1], *James Lowey*[1], *Zixiang Xiong*[2], *and Edward R. Dougherty*[1,2]

[1]Computational Biology Division, Translational Genomics Research Institute, Phoenix, USA
[2]Dept of Electrical and Computer Engineering, Texas A&M University, College Station, USA
Corresponding author:Edward R. Dougherty
Email address: Edward@ece.tamu.edu

March 15, 2006

Contents

1 Synthetic data **3**
1.1 Comparison for Linear Model . . . 3
1.1.1 5-Feature Cases . . . 3
1.1.2 10-Feature Cases . . . 6
1.2 Comparison for Low-Curvature Nonlinear Model . . . 9
1.2.1 5-Feature Cases . . . 9
1.2.2 10-Feature Cases . . . 12
1.3 Comparison for High-Curvature Nonlinear Model . . . 15
1.3.1 5-Feature Cases . . . 15
1.3.2 10-Feature Cases . . . 18
1.4 Comparison for Equal-Mean Model . . . 21
1.4.1 5-Feature Cases . . . 21
1.4.2 10-Feature Cases . . . 24
1.5 Comparison for XOR model . . . 27
1.5.1 5-Feature Cases . . . 27
1.5.2 10-Feature Cases . . . 30
1.6 Comparison for bimodal model . . . 33
1.6.1 5-Feature Cases . . . 33
1.6.2 10-Feature Cases . . . 36

2 Real patient data **39**
2.1 5 features . . . 39
2.2 10 features . . . 40

1 Synthetic data

1.1 Comparison for Linear Model

1.1.1 5-Feature Cases

uncorrelated features

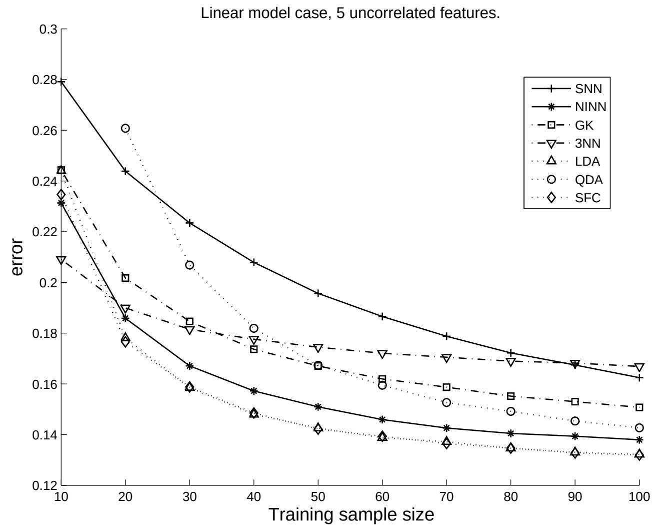

Figure 1: Classification error vs. training sample size for different classifiers.

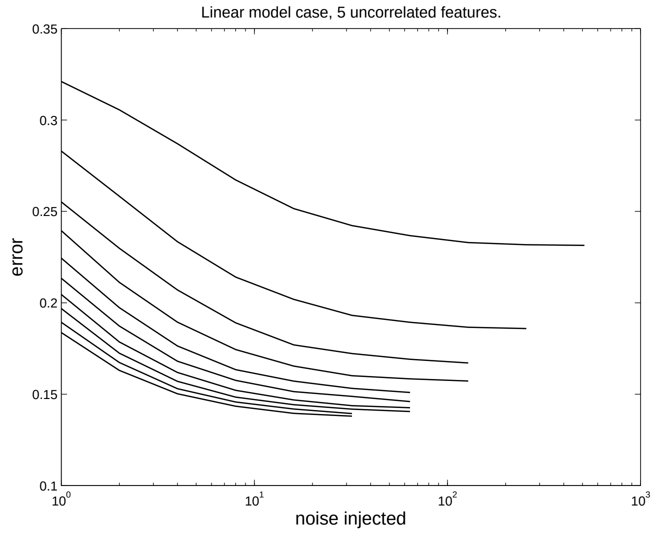

Figure 2: Classification error vs. amount of noise injection. Each line corresponds to a fixed training sample size, from 10 to 100 for every 10 samples. The highest line is 10 training sample case, while the lowest line is the 100 training sample case.

slightly correlated features

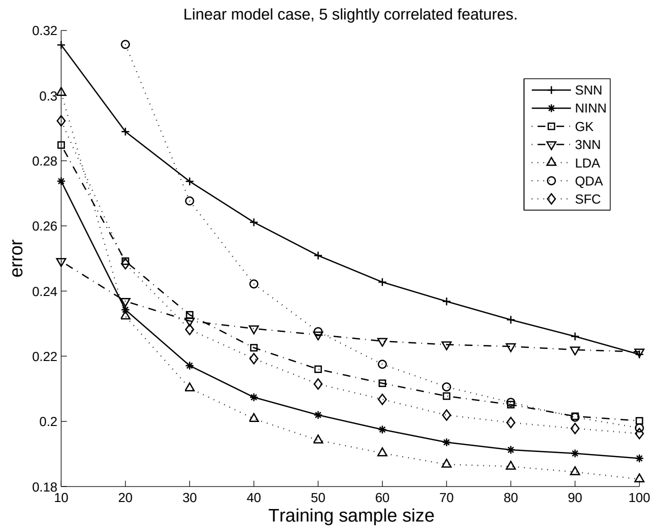

Figure 3: Classification error vs. training sample size for different classifiers.

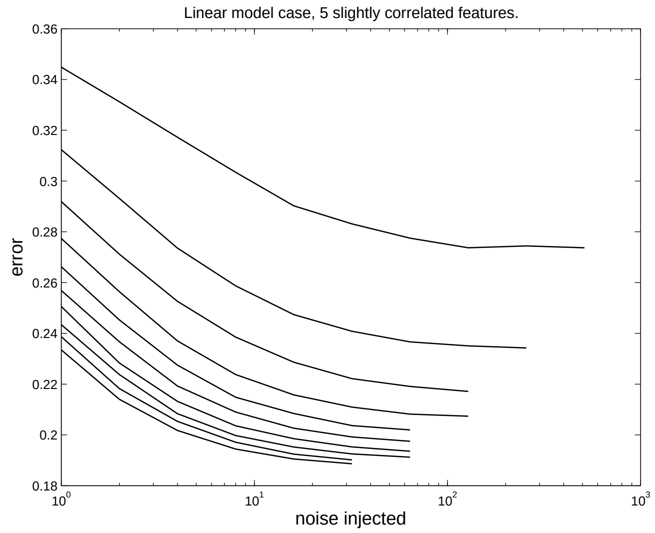

Figure 4: Classification error vs. amount of noise injection. Each line corresponds to a fixed training sample size, from 10 to 100 for every 10 samples. The highest line is 10 training sample case, while the lowest line is the 100 training sample case.

highly correlated features

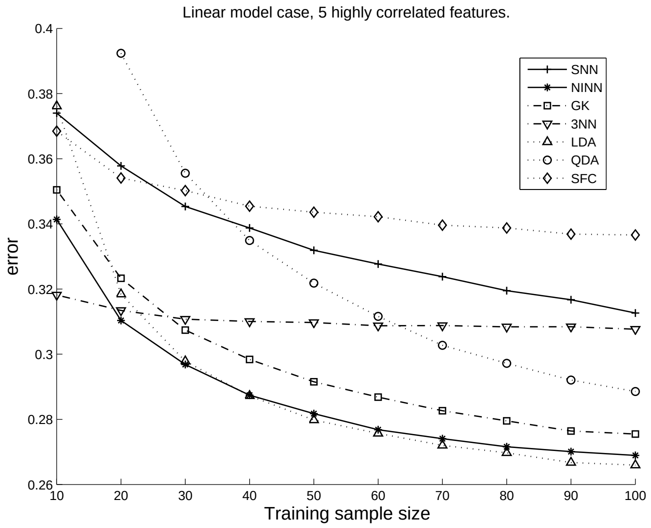

Figure 5: Classification error vs. training sample size for different classifiers.

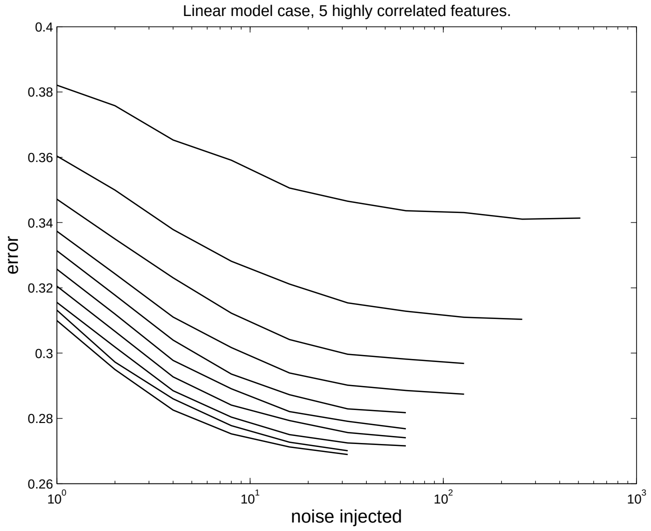

Figure 6: Classification error vs. amount of noise injection. Each line corresponds to a fixed training sample size, from 10 to 100 for every 10 samples. The highest line is 10 training sample case, while the lowest line is the 100 training sample case.

1.1.2 10-Feature Cases

uncorrelated features

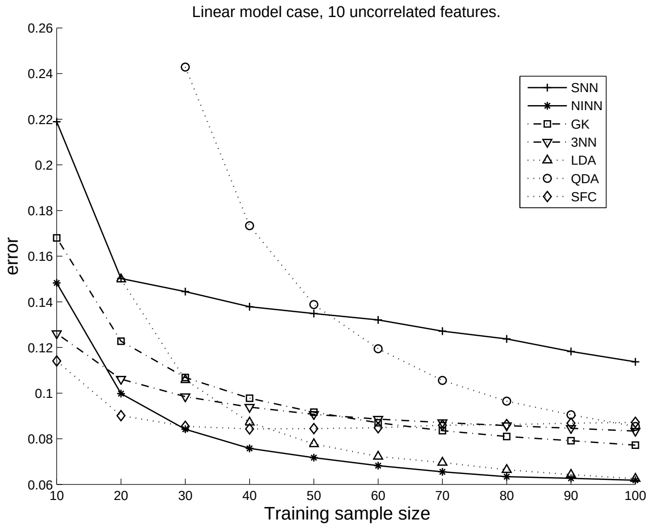

Figure 7: Classification error vs. training sample size for different classifiers.

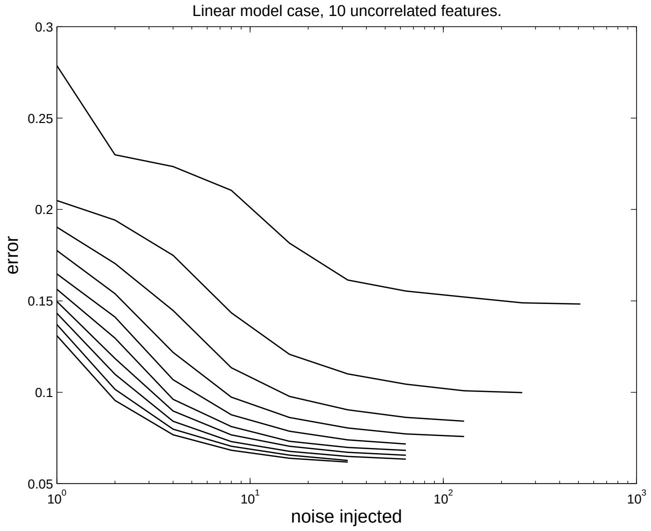

Figure 8: Classification error vs. amount of noise injection. Each line corresponds to a fixed training sample size, from 10 to 100 for every 10 samples. The highest line is 10 training sample case, while the lowest line is the 100 training sample case.

slightly correlated features

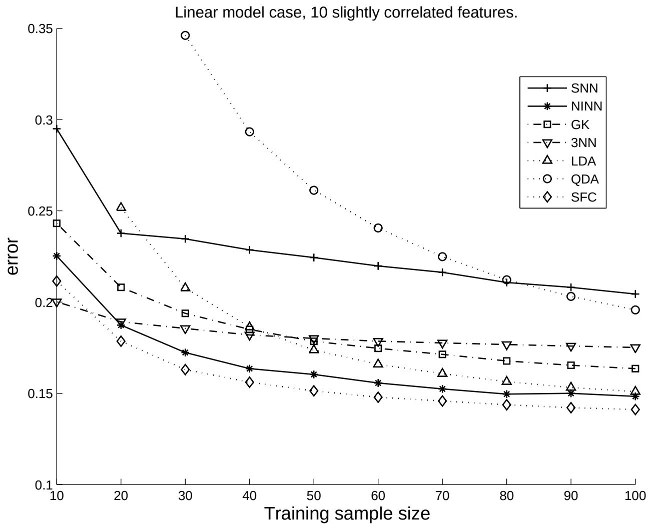

Figure 9: Classification error vs. training sample size for different classifiers.

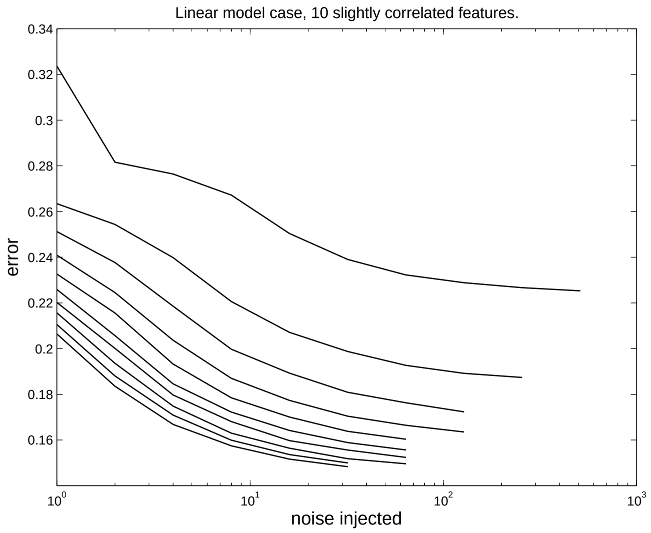

Figure 10: Classification error vs. amount of noise injection. Each line corresponds to a fixed training sample size, from 10 to 100 for every 10 samples. The highest line is 10 training sample case, while the lowest line is the 100 training sample case.

highly correlated features

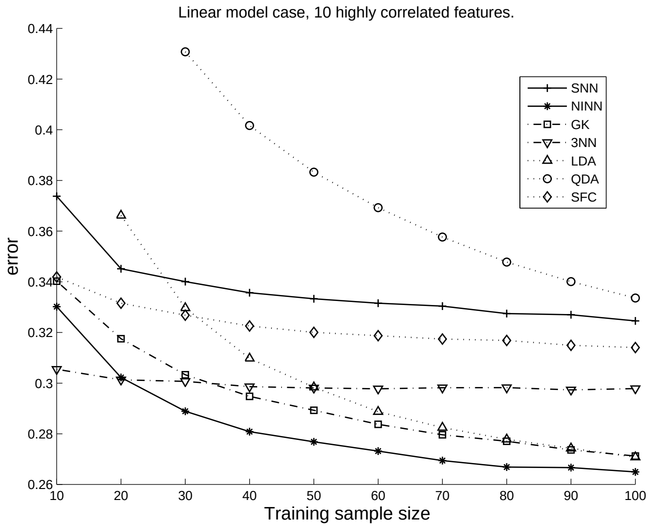

Figure 11: Classification error vs. training sample size for different classifiers.

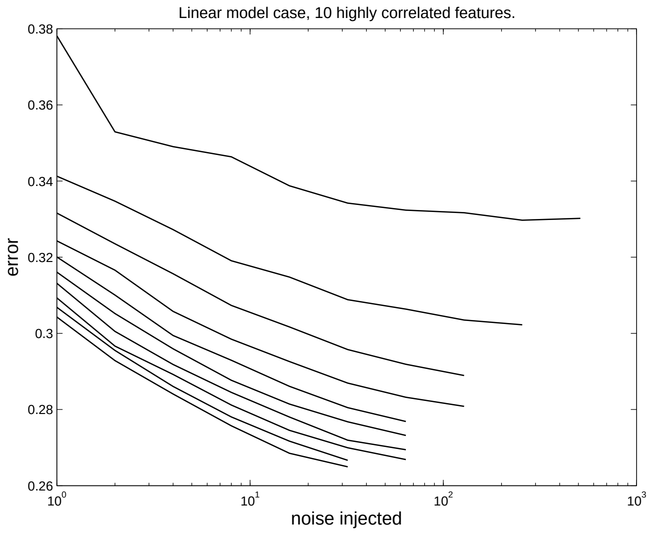

Figure 12: Classification error vs. amount of noise injection. Each line corresponds to a fixed training sample size, from 10 to 100 for every 10 samples. The highest line is 10 training sample case, while the lowest line is the 100 training sample case.

1.2 Comparison for Low-Curvature Nonlinear Model

1.2.1 5-Feature Cases

uncorrelated features

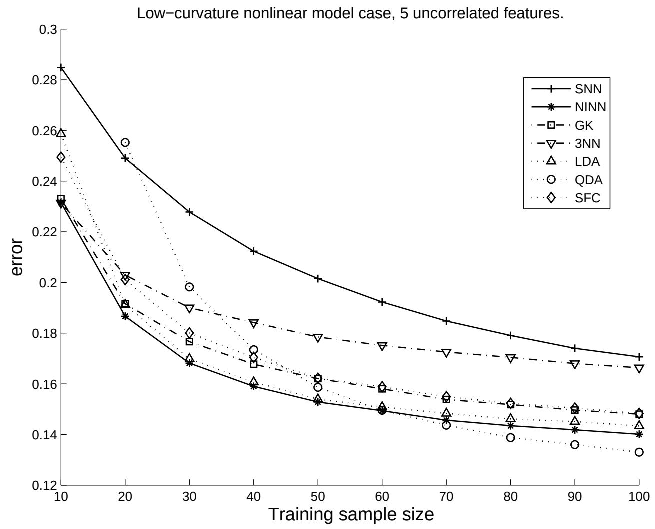

Figure 13: Classification error vs. training sample size for different classifiers.

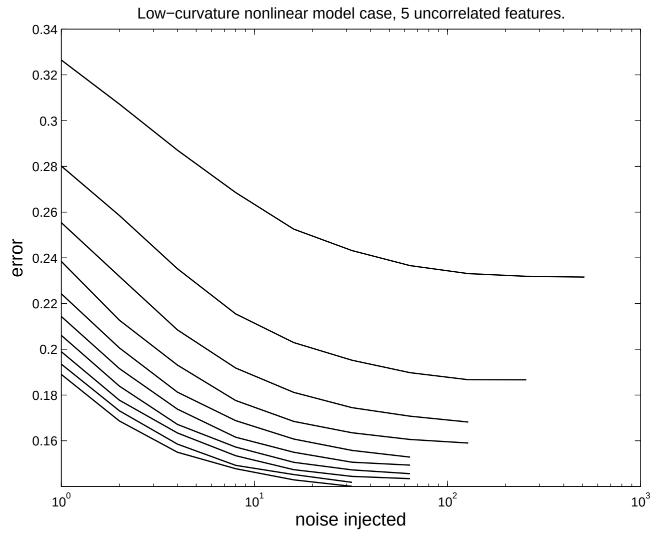

Figure 14: Classification error vs. amount of noise injection. Each line corresponds to a fixed training sample size, from 10 to 100 for every 10 samples. The highest line is 10 training sample case, while the lowest line is the 100 training sample case.

slightly correlated features

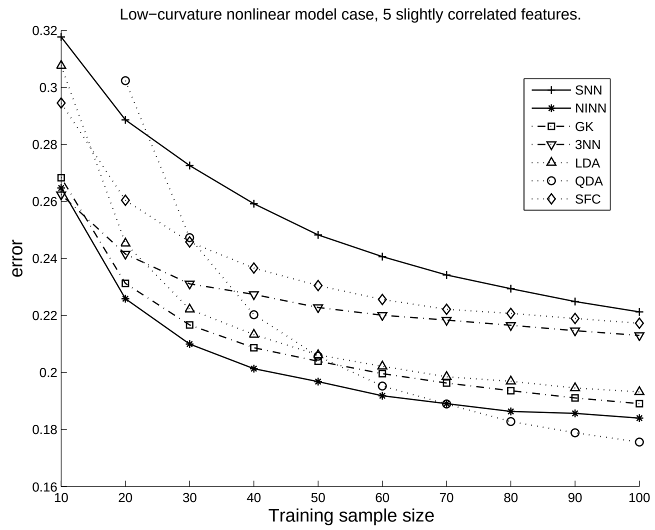

Figure 15: Classification error vs. training sample size for different classifiers.

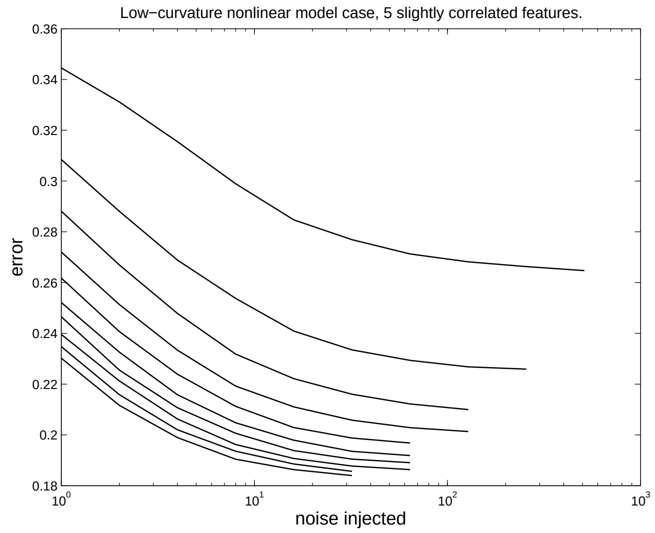

Figure 16: Classification error vs. amount of noise injection. Each line corresponds to a fixed training sample size, from 10 to 100 for every 10 samples. The highest line is 10 training sample case, while the lowest line is the 100 training sample case.

highly correlated features

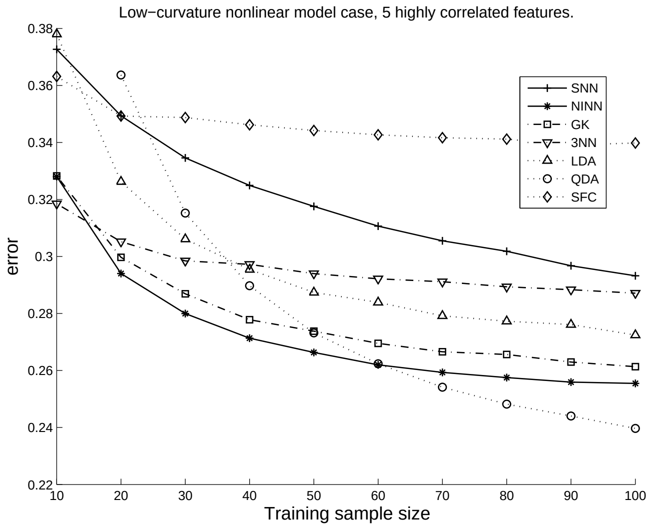

Figure 17: Classification error vs. training sample size for different classifiers.

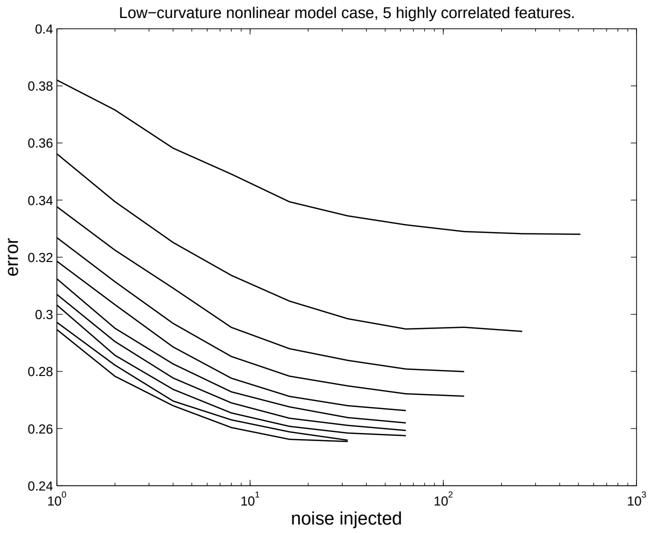

Figure 18: Classification error vs. amount of noise injection. Each line corresponds to a fixed training sample size, from 10 to 100 for every 10 samples. The highest line is 10 training sample case, while the lowest line is the 100 training sample case.

1.2.2 10-Feature Cases

uncorrelated features

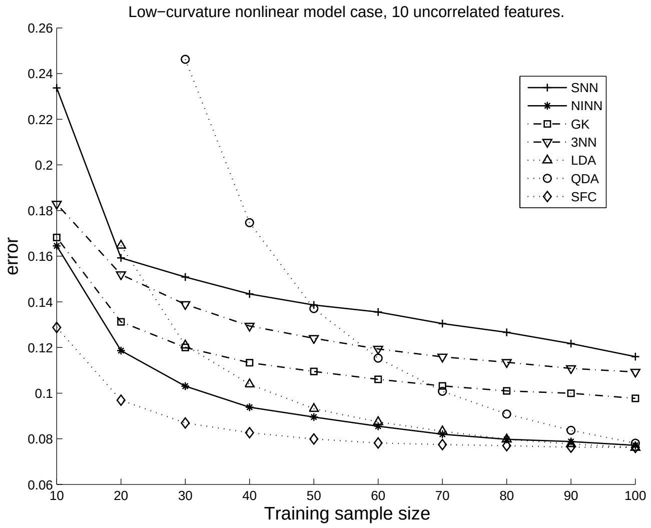

Figure 19: Classification error vs. training sample size for different classifiers.

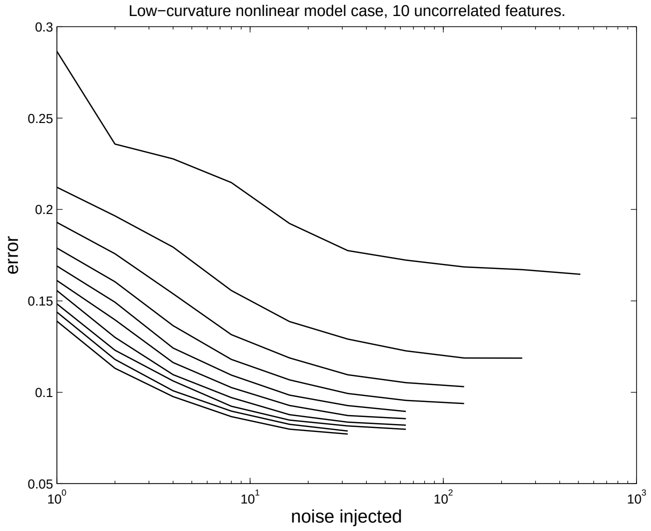

Figure 20: Classification error vs. amount of noise injection. Each line corresponds to a fixed training sample size, from 10 to 100 for every 10 samples. The highest line is 10 training sample case, while the lowest line is the 100 training sample case.

slightly correlated features

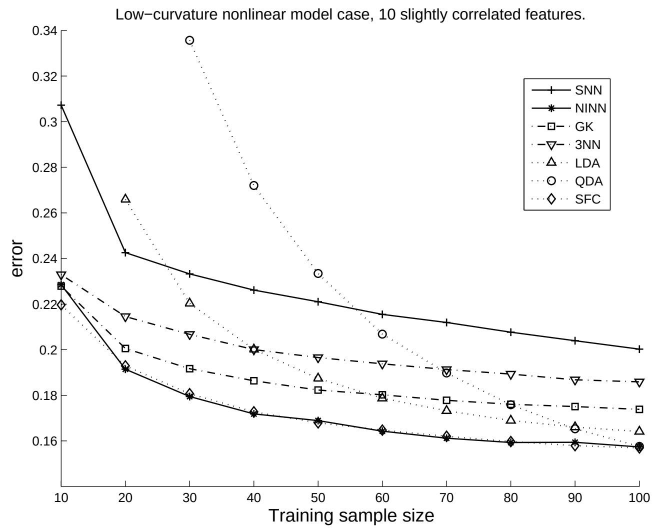

Figure 21: Classification error vs. training sample size for different classifiers.

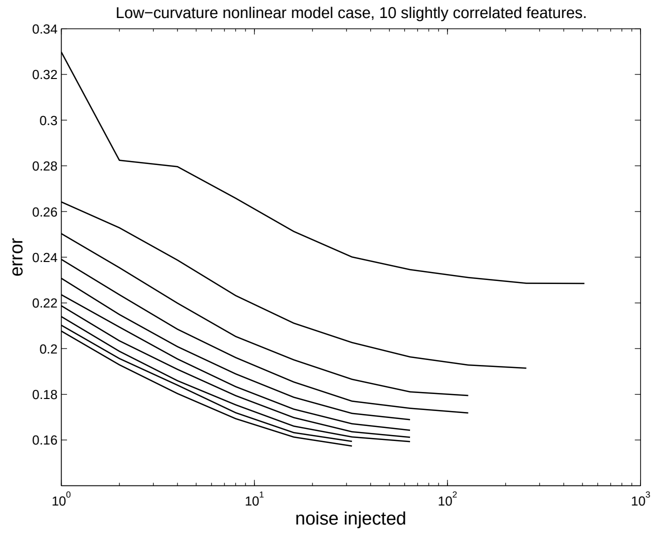

Figure 22: Classification error vs. amount of noise injection. Each line corresponds to a fixed training sample size, from 10 to 100 for every 10 samples. The highest line is 10 training sample case, while the lowest line is the 100 training sample case.

highly correlated features

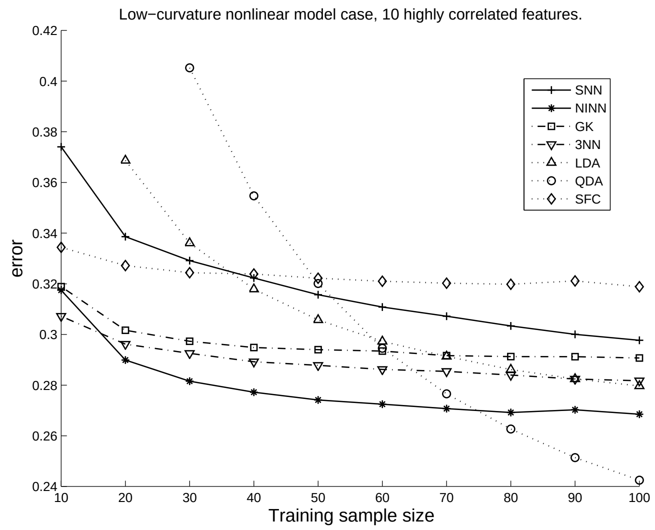

Figure 23: Classification error vs. training sample size for different classifiers.

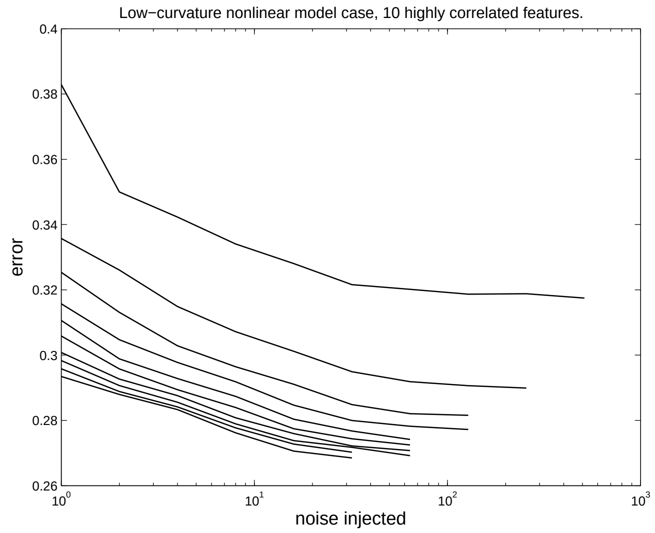

Figure 24: Classification error vs. amount of noise injection. Each line corresponds to a fixed training sample size, from 10 to 100 for every 10 samples. The highest line is 10 training sample case, while the lowest line is the 100 training sample case.

1.3 Comparison for High-Curvature Nonlinear Model

1.3.1 5-Feature Cases

uncorrelated features

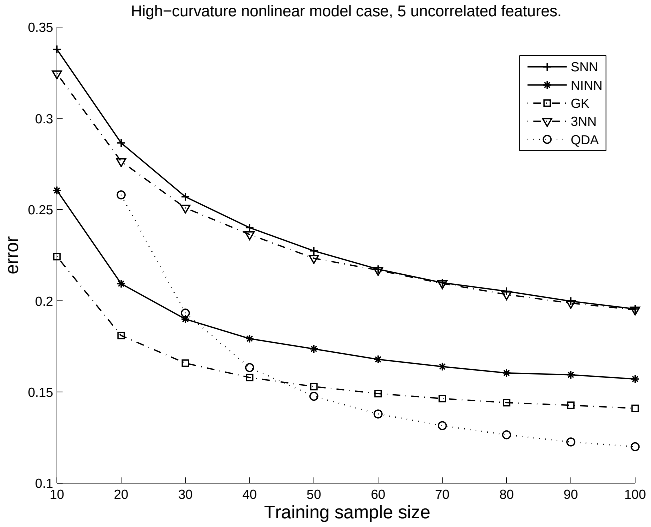

Figure 25: Classification error vs. training sample size for different classifiers.

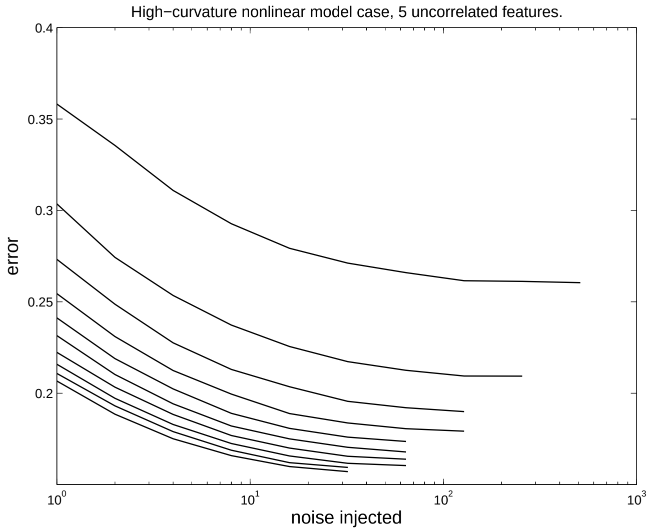

Figure 26: Classification error vs. amount of noise injection. Each line corresponds to a fixed training sample size, from 10 to 100 for every 10 samples. The highest line is 10 training sample case, while the lowest line is the 100 training sample case.

slightly correlated features

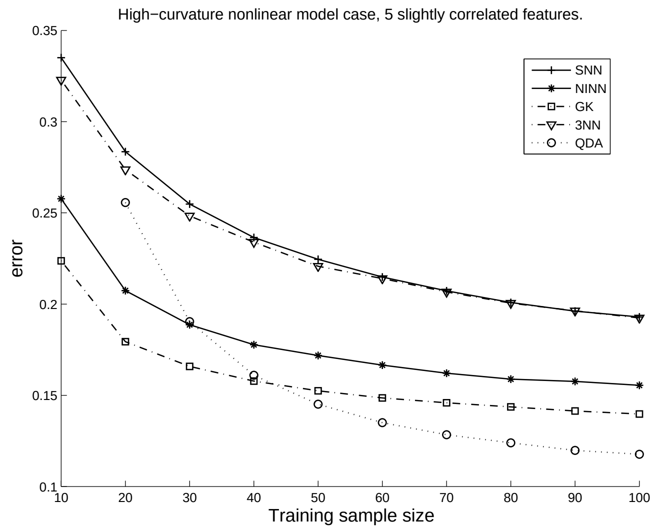

Figure 27: Classification error vs. training sample size for different classifiers.

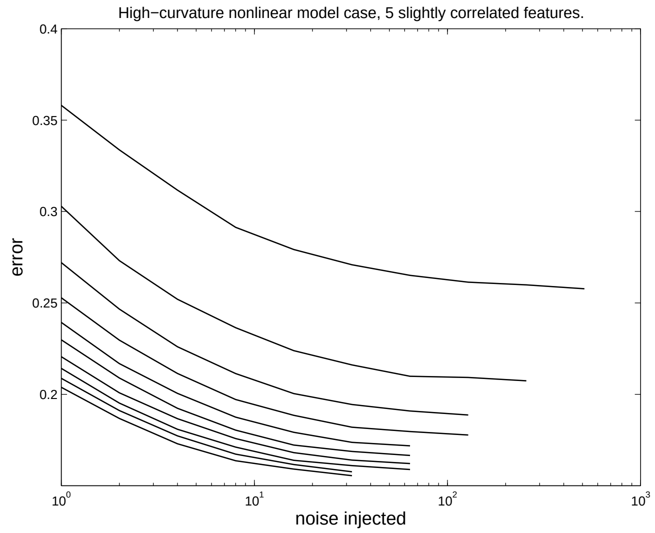

Figure 28: Classification error vs. amount of noise injection. Each line corresponds to a fixed training sample size, from 10 to 100 for every 10 samples. The highest line is 10 training sample case, while the lowest line is the 100 training sample case.

highly correlated features

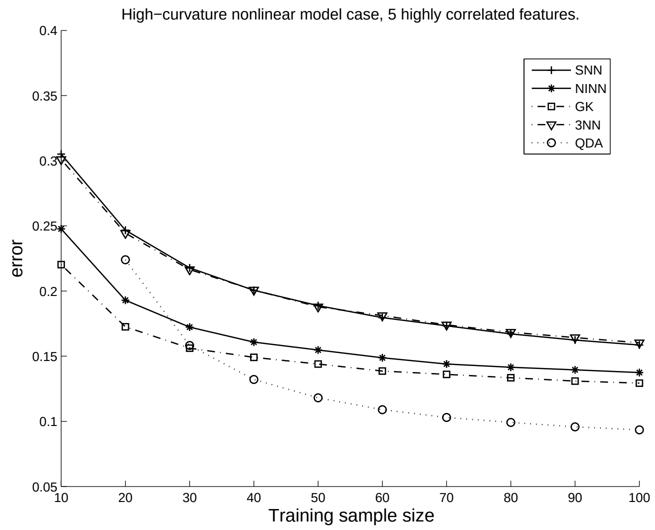

Figure 29: Classification error vs. training sample size for different classifiers.

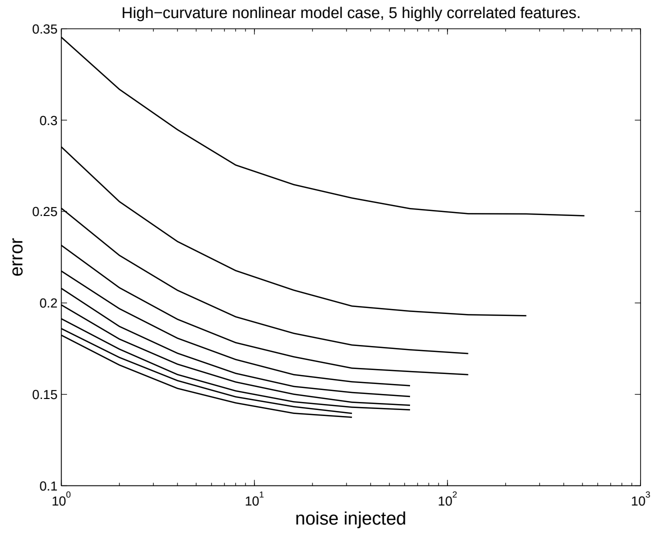

Figure 30: Classification error vs. amount of noise injection. Each line corresponds to a fixed training sample size, from 10 to 100 for every 10 samples. The highest line is 10 training sample case, while the lowest line is the 100 training sample case.

1.3.2 10-Feature Cases

uncorrelated features

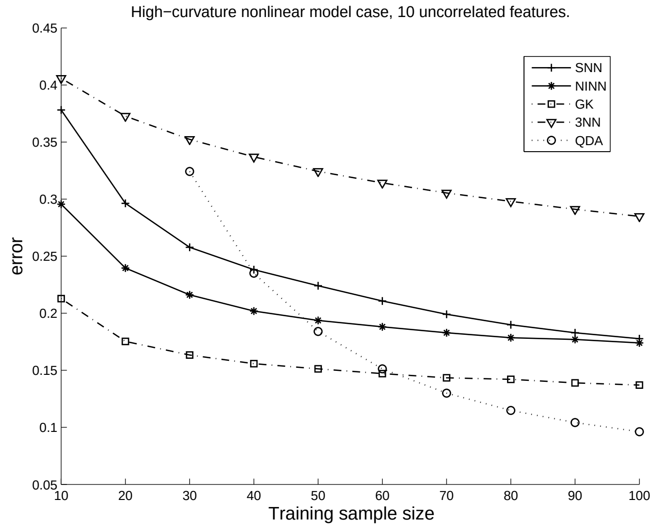

Figure 31: Classification error vs. training sample size for different classifiers.

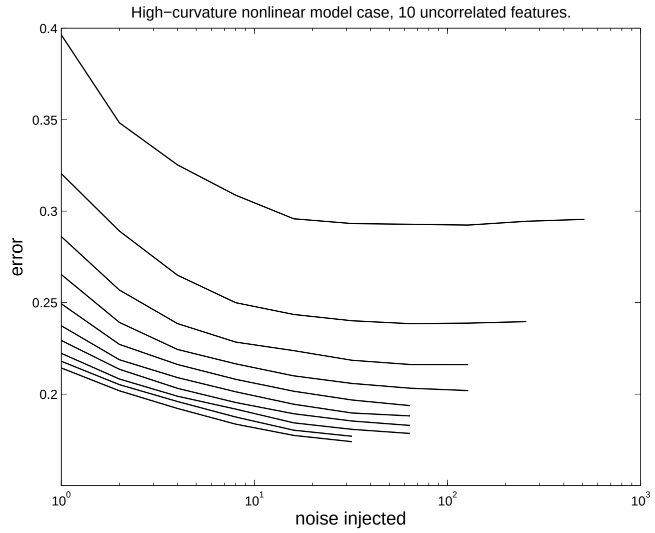

Figure 32: Classification error vs. amount of noise injection. Each line corresponds to a fixed training sample size, from 10 to 100 for every 10 samples. The highest line is 10 training sample case, while the lowest line is the 100 training sample case.

slightly correlated features

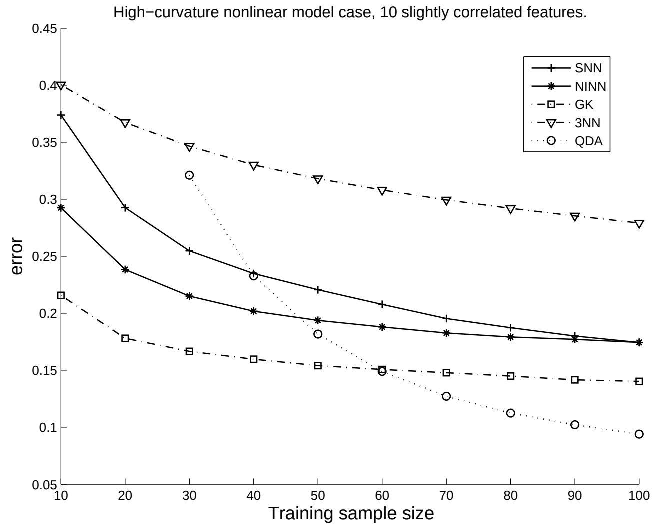

Figure 33: Classification error vs. training sample size for different classifiers.

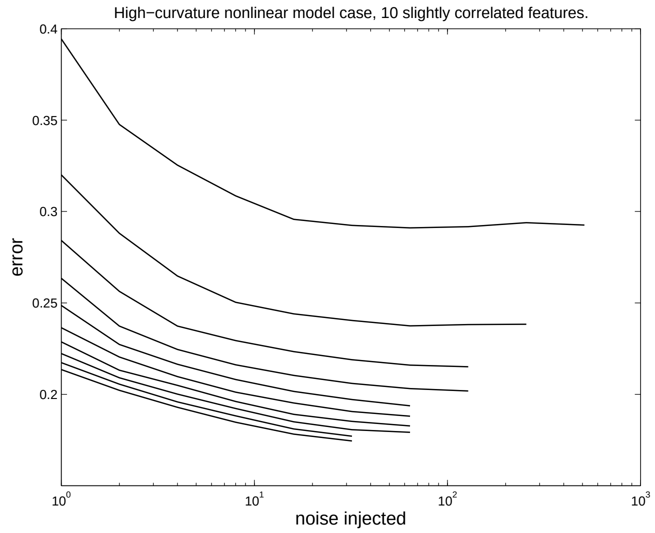

Figure 34: Classification error vs. amount of noise injection. Each line corresponds to a fixed training sample size, from 10 to 100 for every 10 samples. The highest line is 10 training sample case, while the lowest line is the 100 training sample case.

highly correlated features

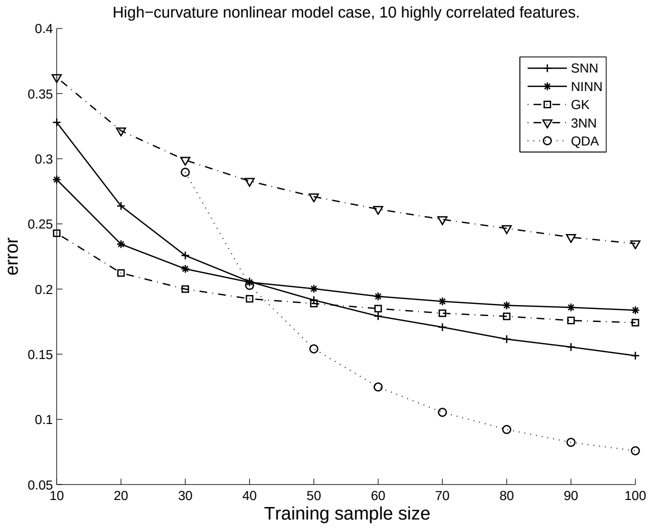

Figure 35: Classification error vs. training sample size for different classifiers.

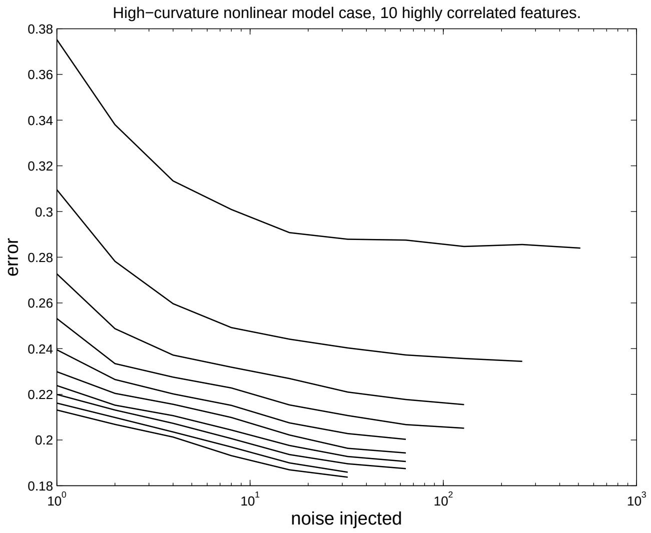

Figure 36: Classification error vs. amount of noise injection. Each line corresponds to a fixed training sample size, from 10 to 100 for every 10 samples. The highest line is 10 training sample case, while the lowest line is the 100 training sample case.

1.4 Comparison for Equal-Mean Model

1.4.1 5-Feature Cases

uncorrelated features

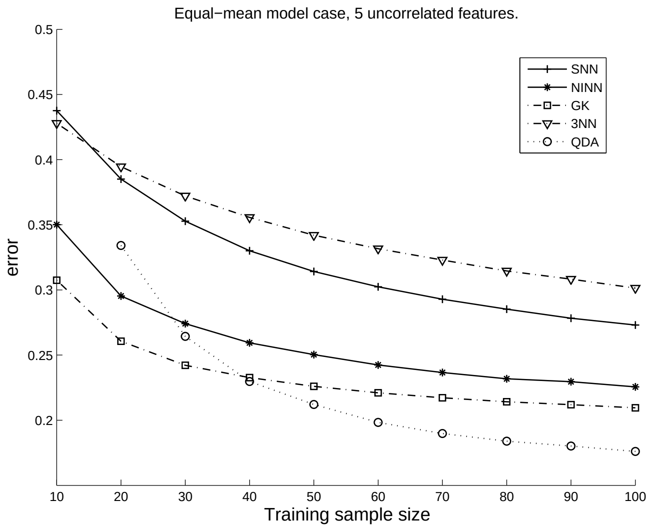

Figure 37: Classification error vs. training sample size for different classifiers.

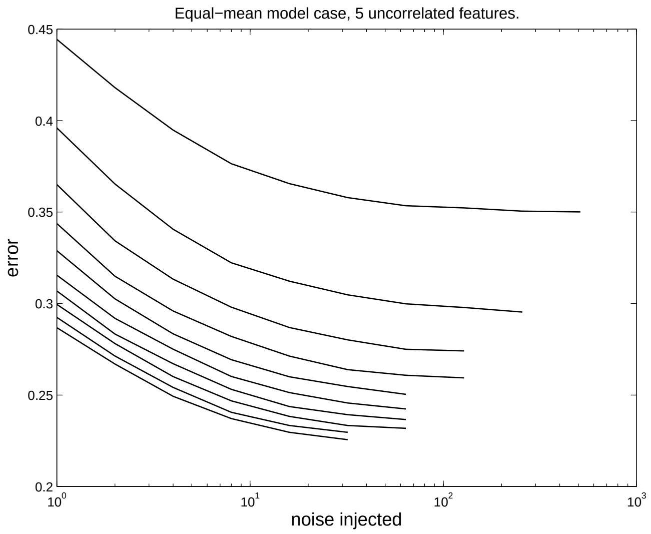

Figure 38: Classification error vs. amount of noise injection. Each line corresponds to a fixed training sample size, from 10 to 100 for every 10 samples. The highest line is 10 training sample case, while the lowest line is the 100 training sample case.

slightly correlated features

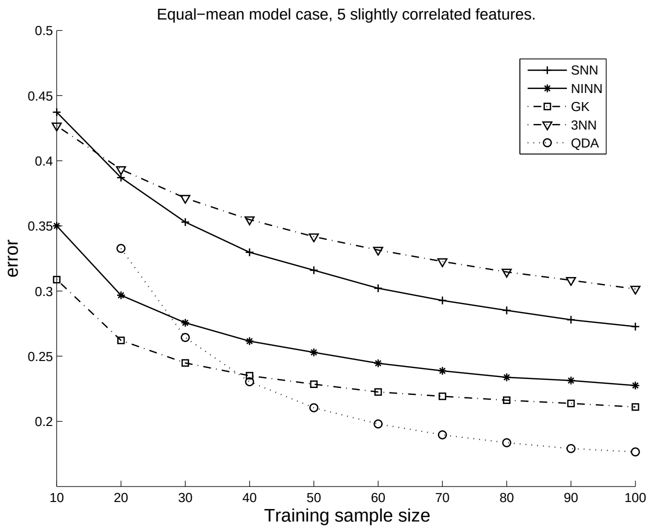

Figure 39: Classification error vs. training sample size for different classifiers.

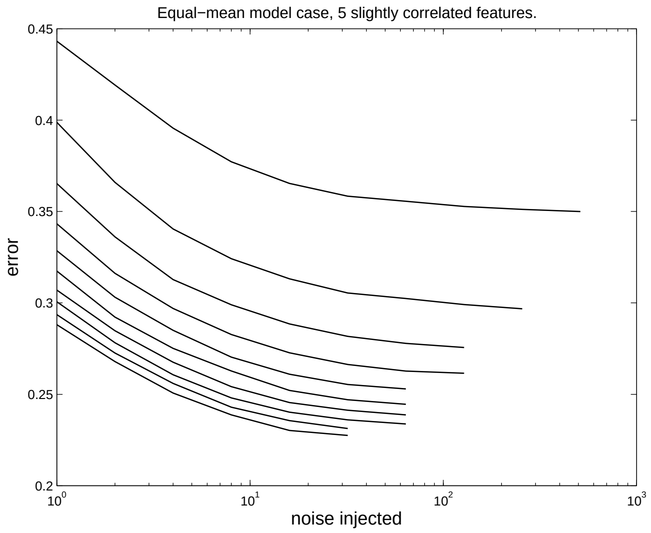

Figure 40: Classification error vs. amount of noise injection. Each line corresponds to a fixed training sample size, from 10 to 100 for every 10 samples. The highest line is 10 training sample case, while the lowest line is the 100 training sample case.

highly correlated features

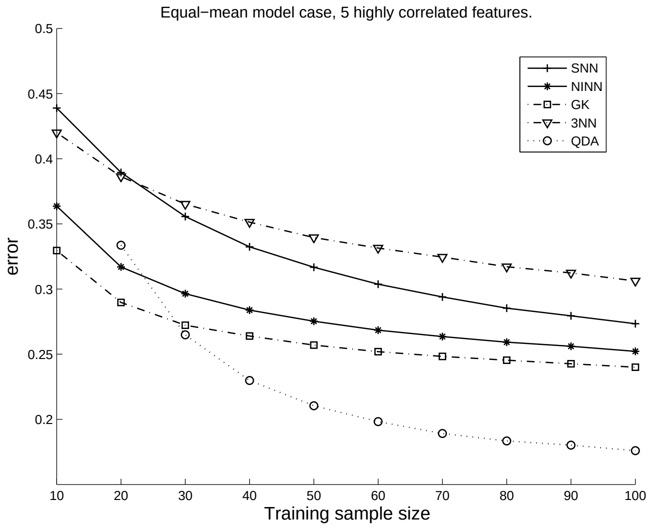

Figure 41: Classification error vs. training sample size for different classifiers.

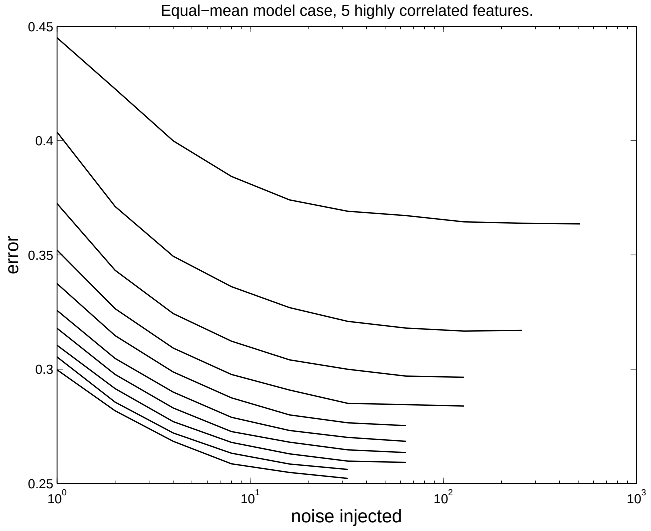

Figure 42: Classification error vs. amount of noise injection. Each line corresponds to a fixed training sample size, from 10 to 100 for every 10 samples. The highest line is 10 training sample case, while the lowest line is the 100 training sample case.

1.4.2 10-Feature Cases

uncorrelated features

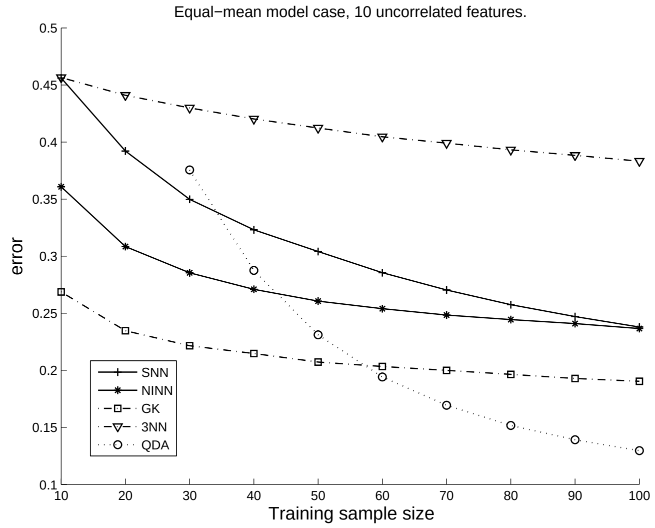

Figure 43: Classification error vs. training sample size for different classifiers.

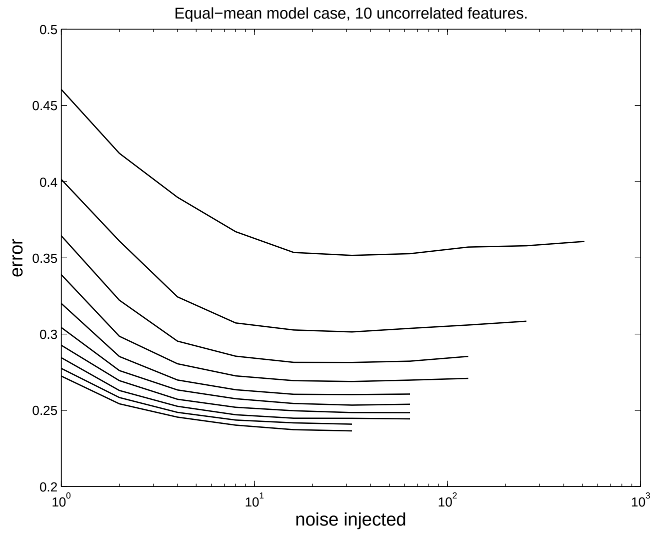

Figure 44: Classification error vs. amount of noise injection. Each line corresponds to a fixed training sample size, from 10 to 100 for every 10 samples. The highest line is 10 training sample case, while the lowest line is the 100 training sample case.

slightly correlated features

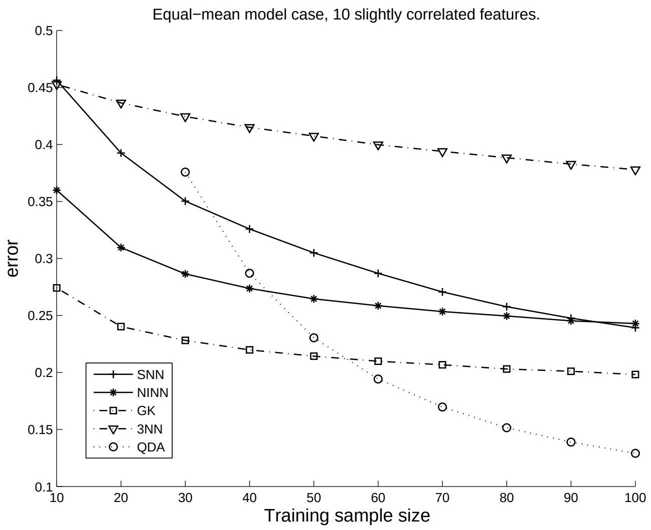

Figure 45: Classification error vs. training sample size for different classifiers.

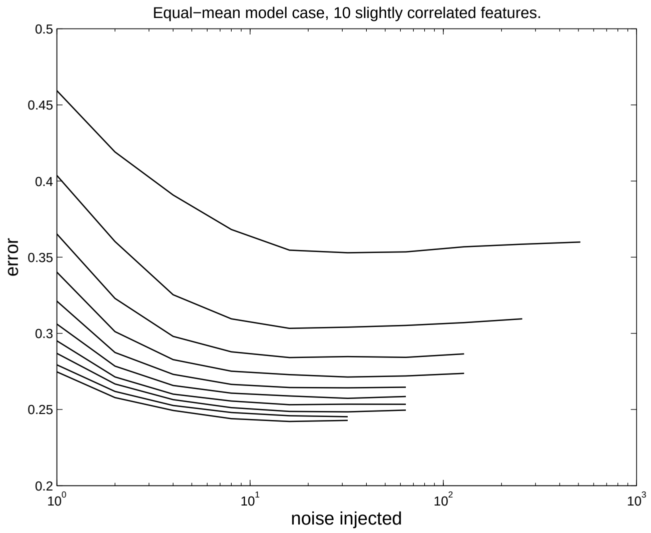

Figure 46: Classification error vs. amount of noise injection. Each line corresponds to a fixed training sample size, from 10 to 100 for every 10 samples. The highest line is 10 training sample case, while the lowest line is the 100 training sample case.

highly correlated features

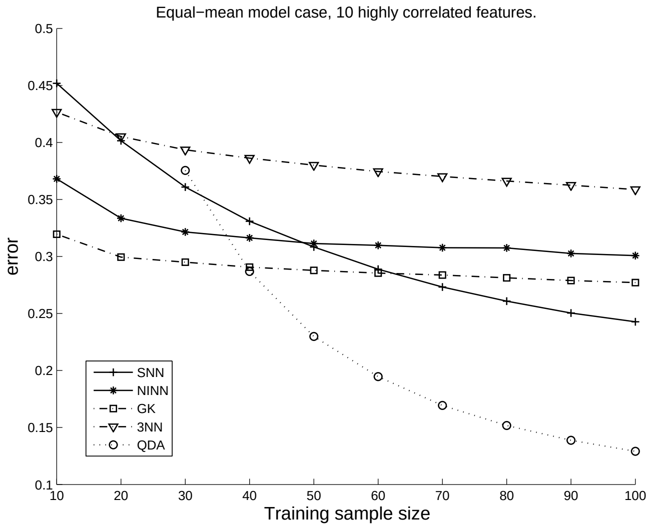

Figure 47: Classification error vs. training sample size for different classifiers.

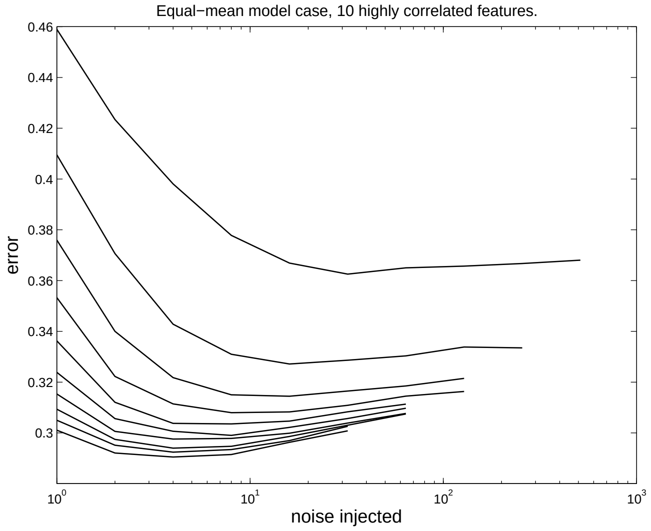

Figure 48: Classification error vs. amount of noise injection. Each line corresponds to a fixed training sample size, from 10 to 100 for every 10 samples. The highest line is 10 training sample case, while the lowest line is the 100 training sample case.

1.5 Comparison for XOR model

1.5.1 5-Feature Cases

uncorrelated features

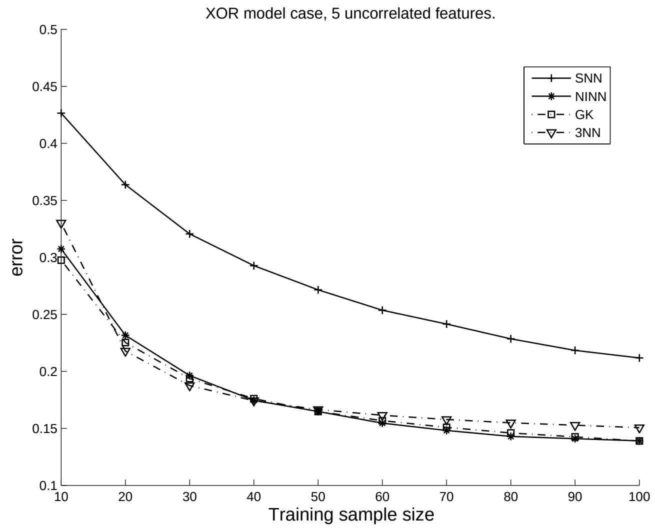

Figure 49: Classification error vs. training sample size for different classifiers.

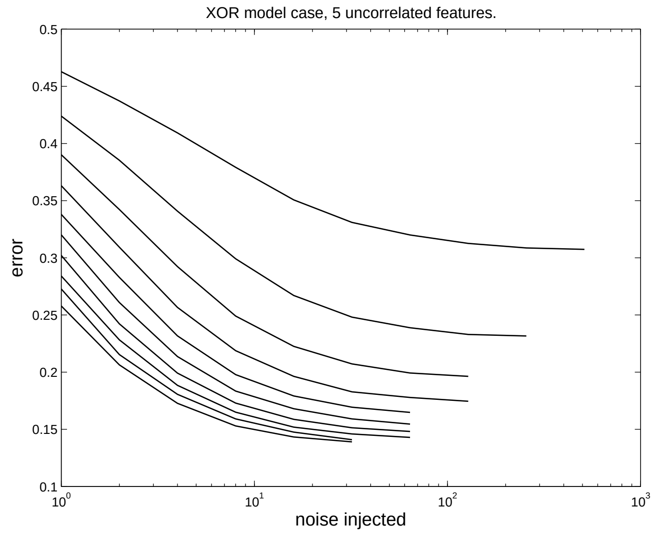

Figure 50: Classification error vs. amount of noise injection. Each line corresponds to a fixed training sample size, from 10 to 100 for every 10 samples. The highest line is 10 training sample case, while the lowest line is the 100 training sample case.

slightly correlated features

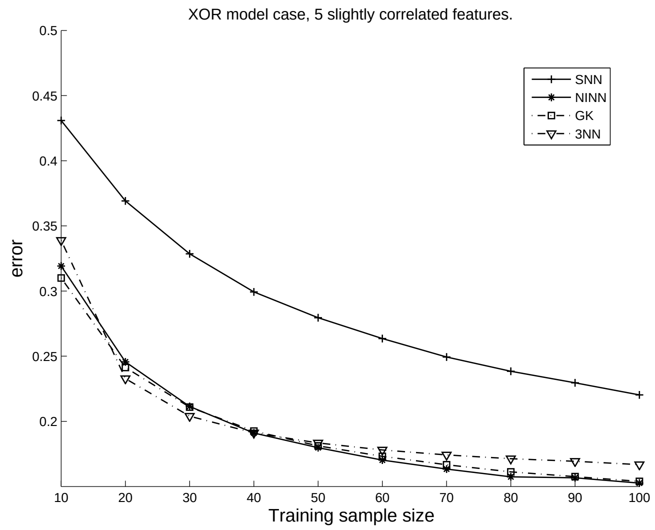

Figure 51: Classification error vs. training sample size for different classifiers.

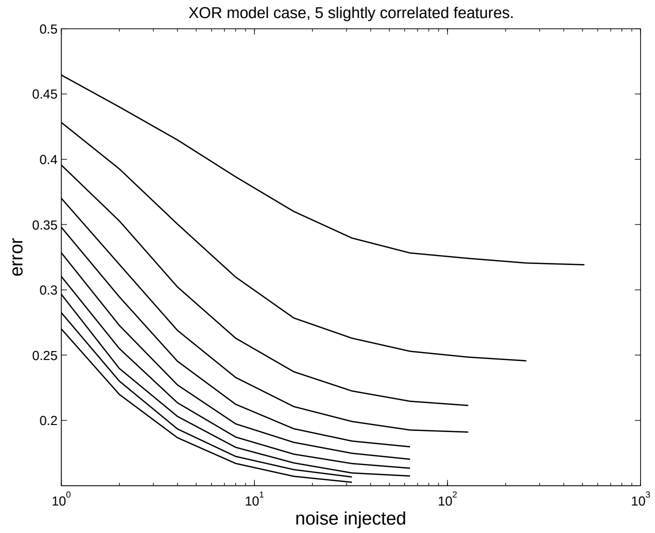

Figure 52: Classification error vs. amount of noise injection. Each line corresponds to a fixed training sample size, from 10 to 100 for every 10 samples. The highest line is 10 training sample case, while the lowest line is the 100 training sample case.

highly correlated features

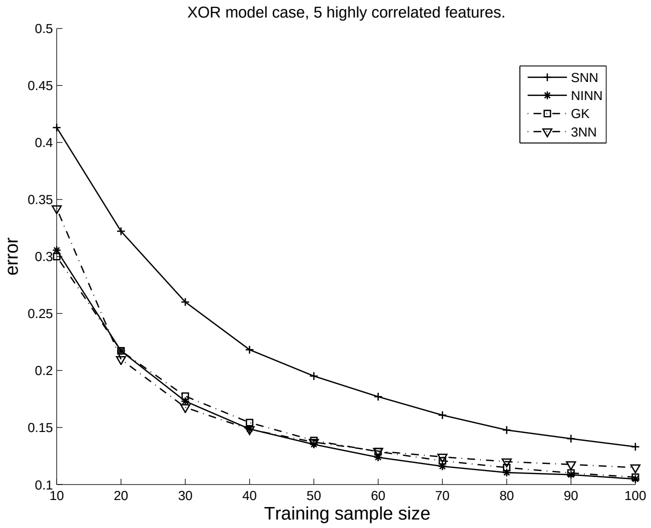

Figure 53: Classification error vs. training sample size for different classifiers.

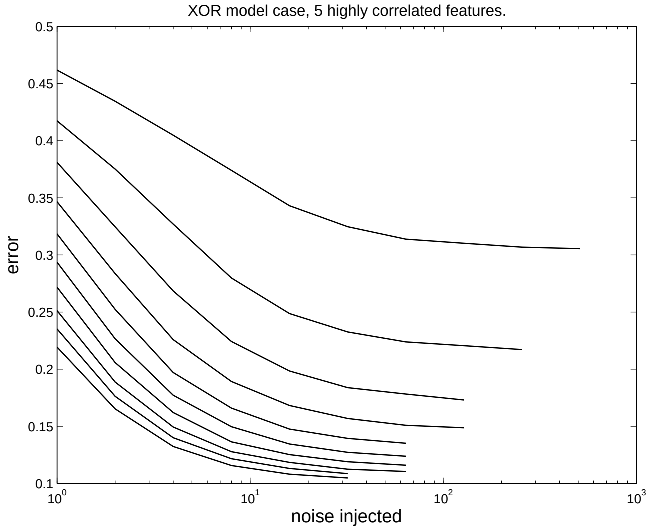

Figure 54: Classification error vs. amount of noise injection. Each line corresponds to a fixed training sample size, from 10 to 100 for every 10 samples. The highest line is 10 training sample case, while the lowest line is the 100 training sample case.

1.5.2 10-Feature Cases

uncorrelated features

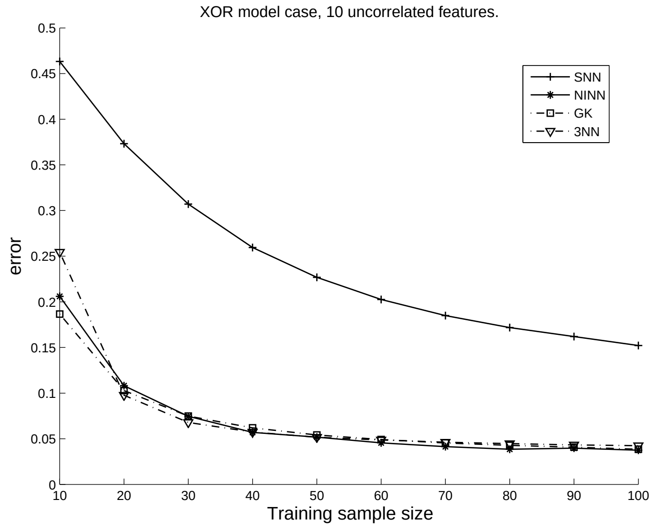

Figure 55: Classification error vs. training sample size for different classifiers.

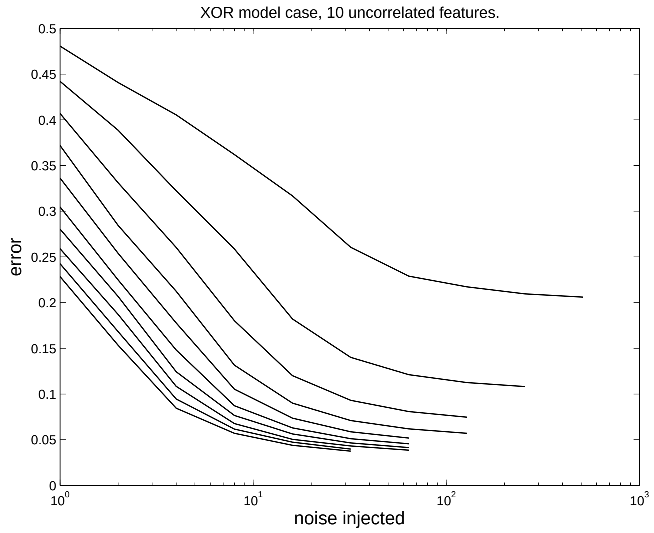

Figure 56: Classification error vs. amount of noise injection. Each line corresponds to a fixed training sample size, from 10 to 100 for every 10 samples. The highest line is 10 training sample case, while the lowest line is the 100 training sample case.

slightly correlated features

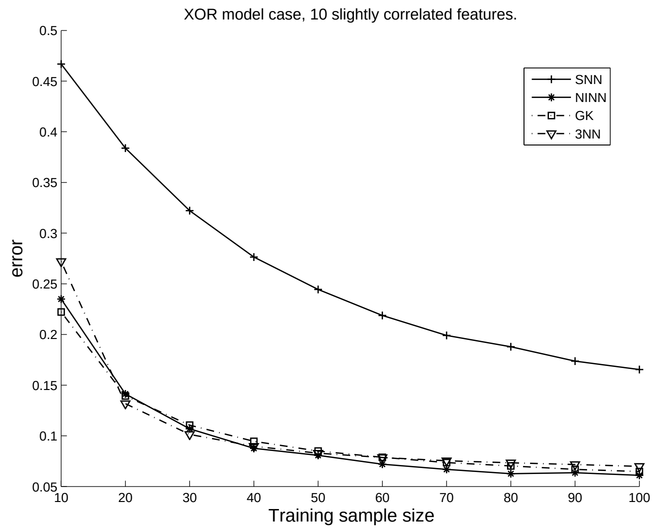

Figure 57: Classification error vs. training sample size for different classifiers.

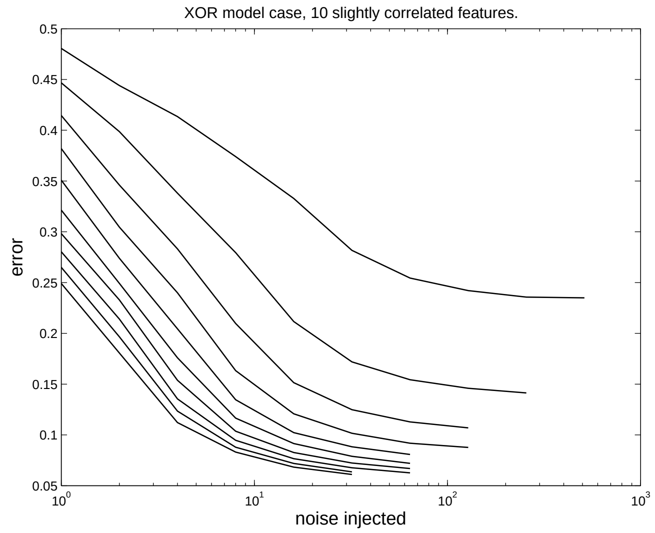

Figure 58: Classification error vs. amount of noise injection. Each line corresponds to a fixed training sample size, from 10 to 100 for every 10 samples. The highest line is 10 training sample case, while the lowest line is the 100 training sample case.

highly correlated features

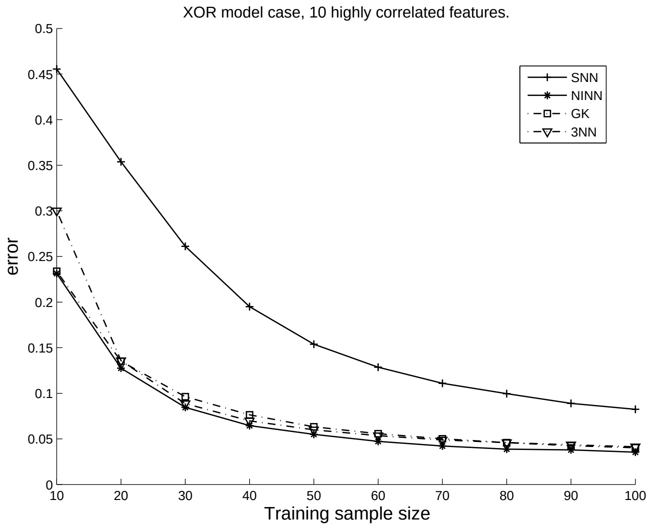

Figure 59: Classification error vs. training sample size for different classifiers.

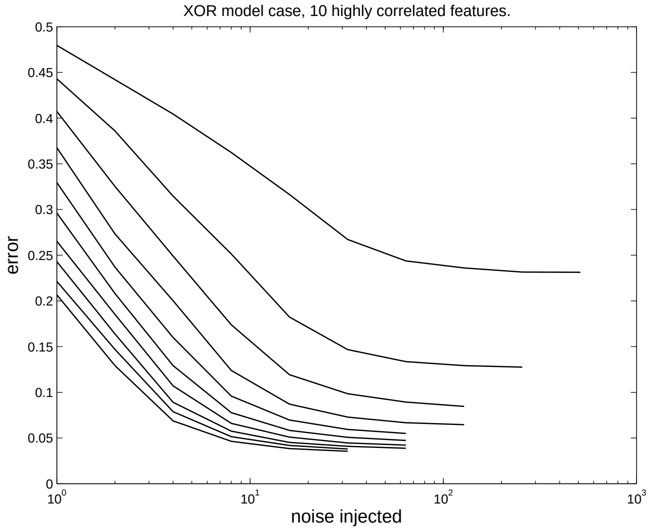

Figure 60: Classification error vs. amount of noise injection. Each line corresponds to a fixed training sample size, from 10 to 100 for every 10 samples. The highest line is 10 training sample case, while the lowest line is the 100 training sample case.

1.6 Comparison for bimodal model

1.6.1 5-Feature Cases

uncorrelated features

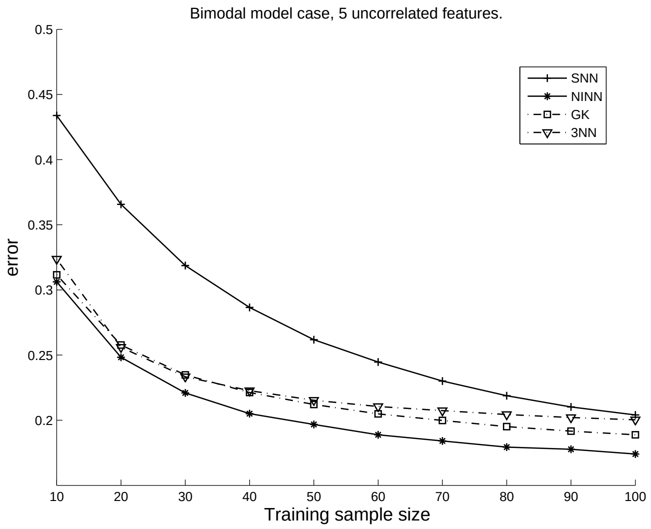

Figure 61: Classification error vs. training sample size for different classifiers.

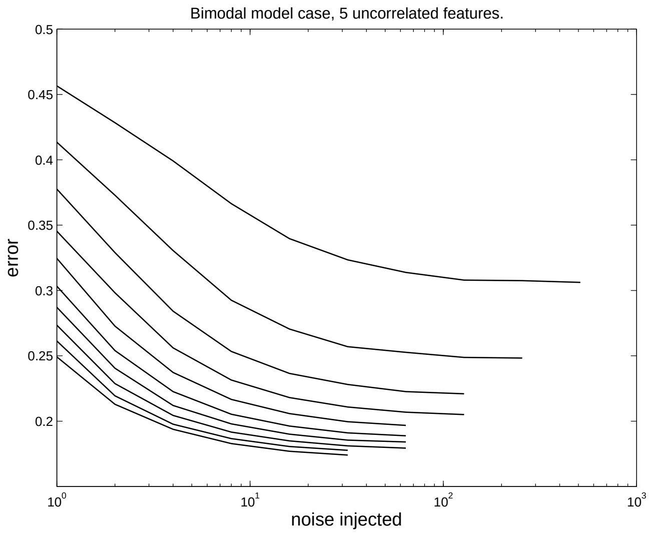

Figure 62: Classification error vs. amount of noise injection. Each line corresponds to a fixed training sample size, from 10 to 100 for every 10 samples. The highest line is 10 training sample case, while the lowest line is the 100 training sample case.

slightly correlated features

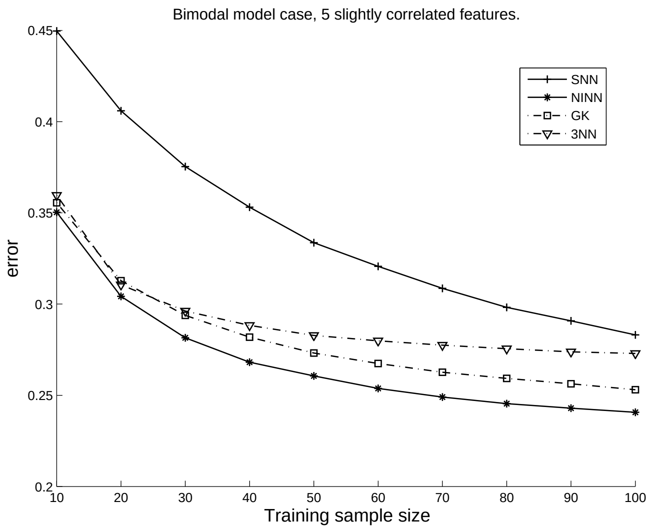

Figure 63: Classification error vs. training sample size for different classifiers.

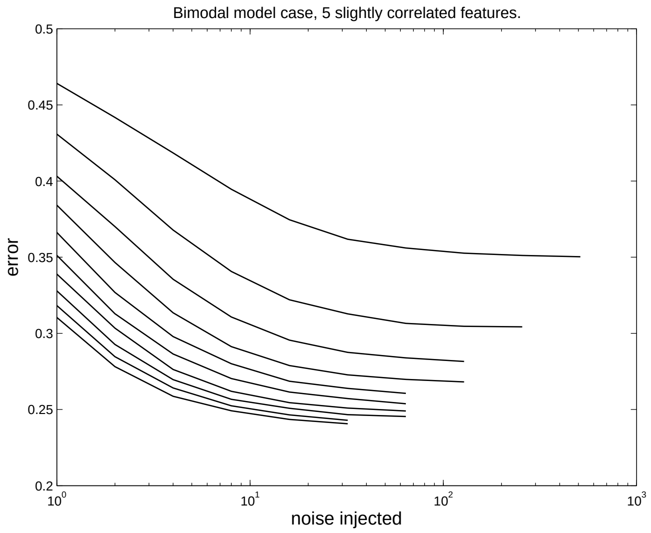

Figure 64: Classification error vs. amount of noise injection. Each line corresponds to a fixed training sample size, from 10 to 100 for every 10 samples. The highest line is 10 training sample case, while the lowest line is the 100 training sample case.

highly correlated features

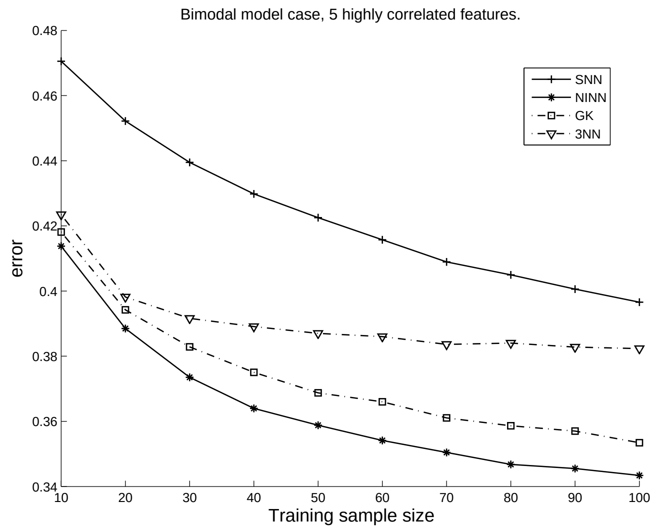

Figure 65: Classification error vs. training sample size for different classifiers.

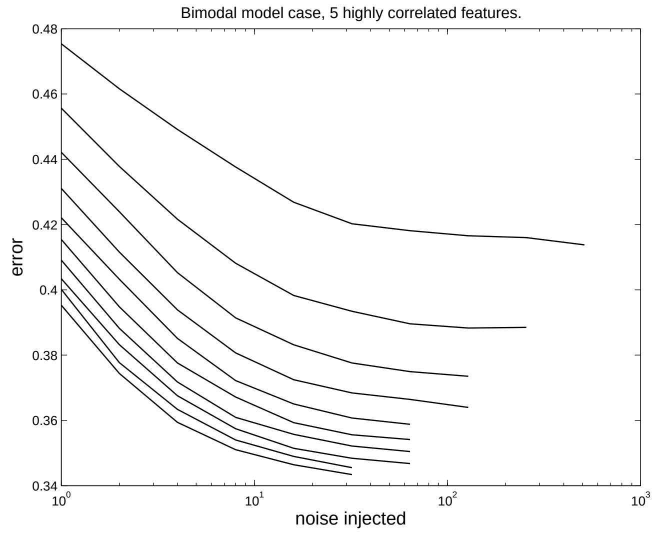

Figure 66: Classification error vs. amount of noise injection. Each line corresponds to a fixed training sample size, from 10 to 100 for every 10 samples. The highest line is 10 training sample case, while the lowest line is the 100 training sample case.

1.6.2 10-Feature Cases

uncorrelated features

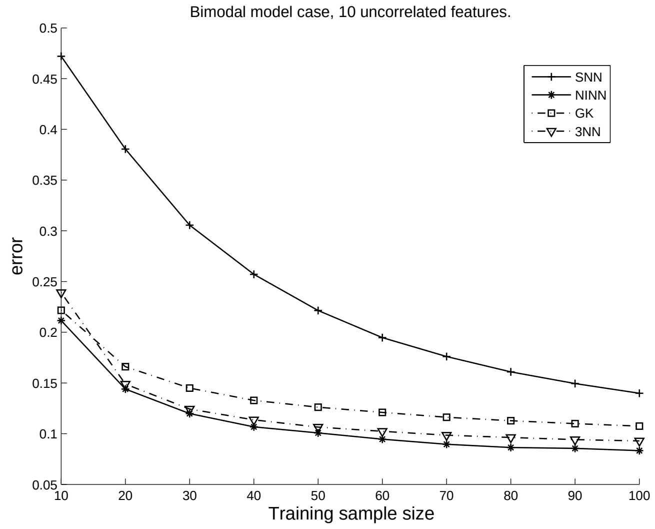

Figure 67: Classification error vs. training sample size for different classifiers.

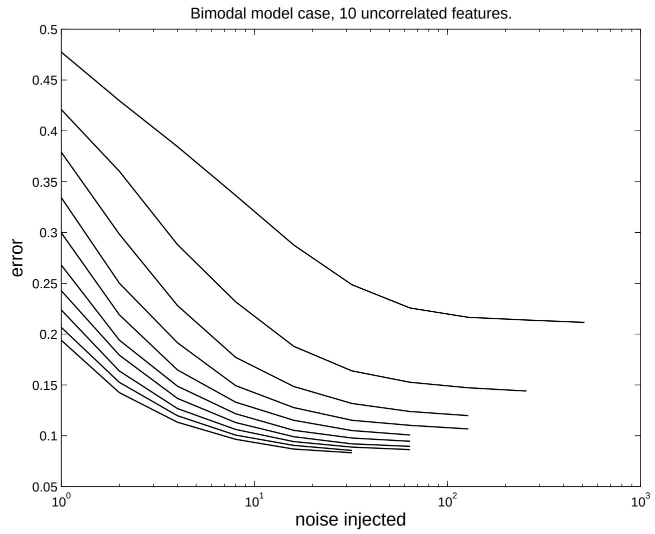

Figure 68: Classification error vs. amount of noise injection. Each line corresponds to a fixed training sample size, from 10 to 100 for every 10 samples. The highest line is 10 training sample case, while the lowest line is the 100 training sample case.

slightly correlated features

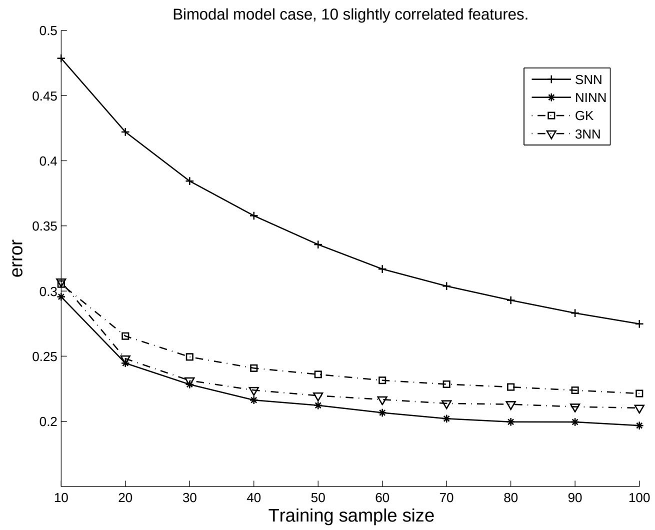

Figure 69: Classification error vs. training sample size for different classifiers.

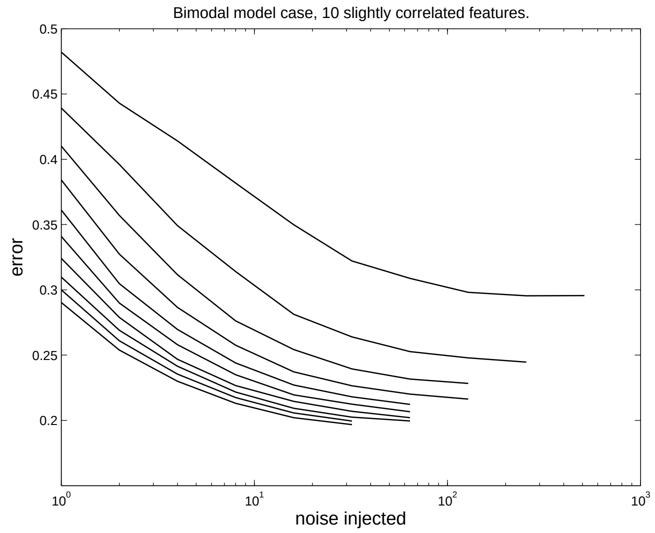

Figure 70: Classification error vs. amount of noise injection. Each line corresponds to a fixed training sample size, from 10 to 100 for every 10 samples. The highest line is 10 training sample case, while the lowest line is the 100 training sample case.

highly correlated features

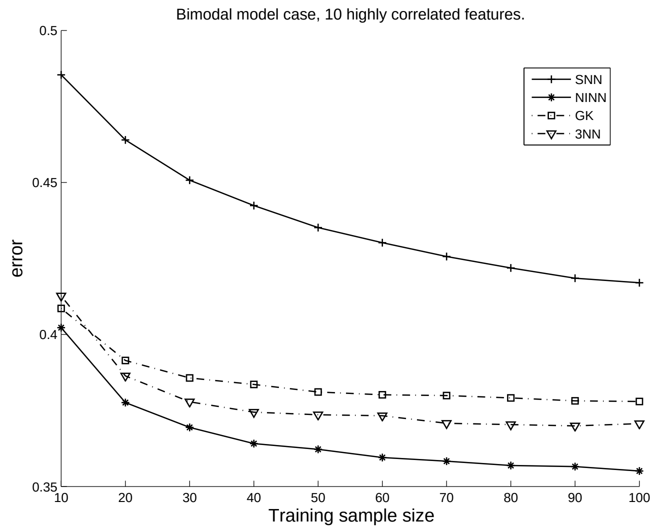

Figure 71: Classification error vs. training sample size for different classifiers.

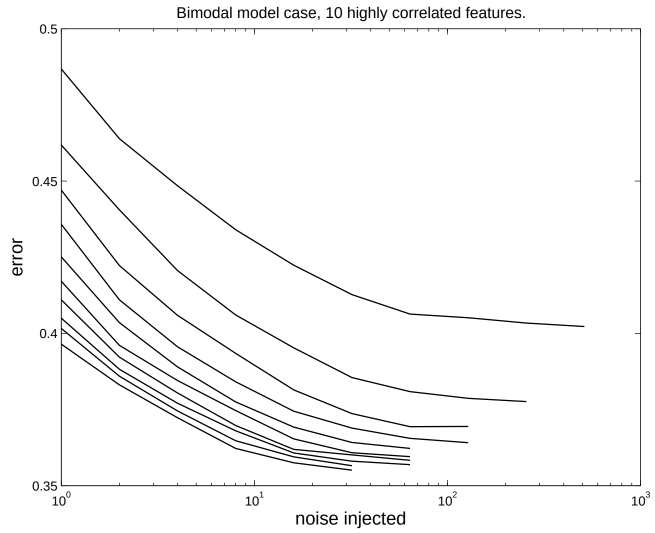

Figure 72: Classification error vs. amount of noise injection. Each line corresponds to a fixed training sample size, from 10 to 100 for every 10 samples. The highest line is 10 training sample case, while the lowest line is the 100 training sample case.

2 Real patient data

2.1 5 features

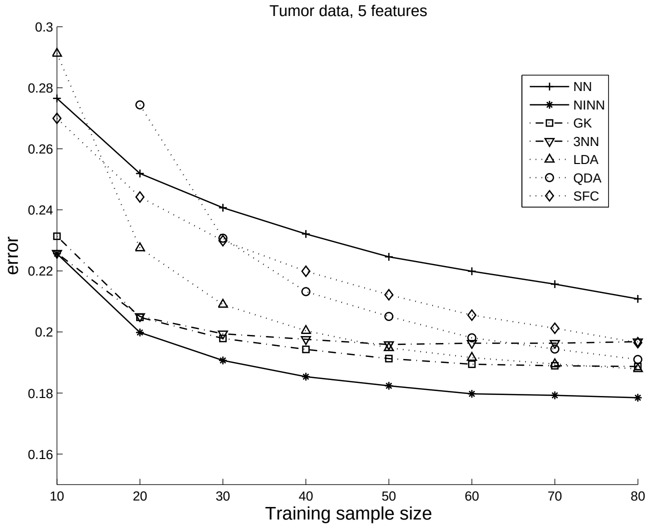

Figure 73: Classification error vs. training sample size for different classifiers.

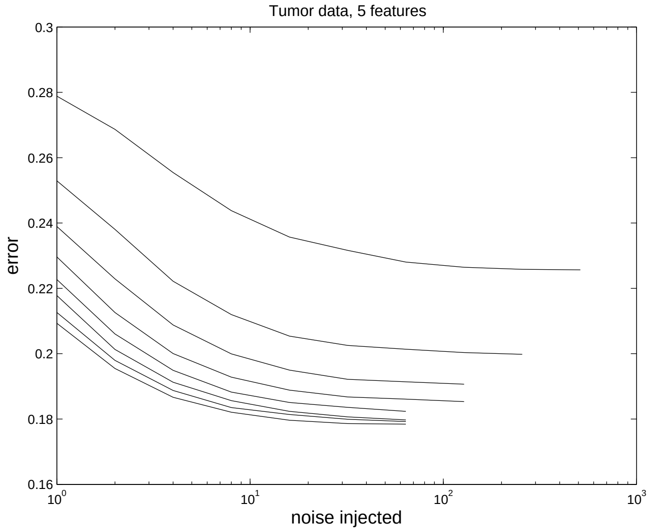

Figure 74: Classification results of noise injection.

2.2 10 features

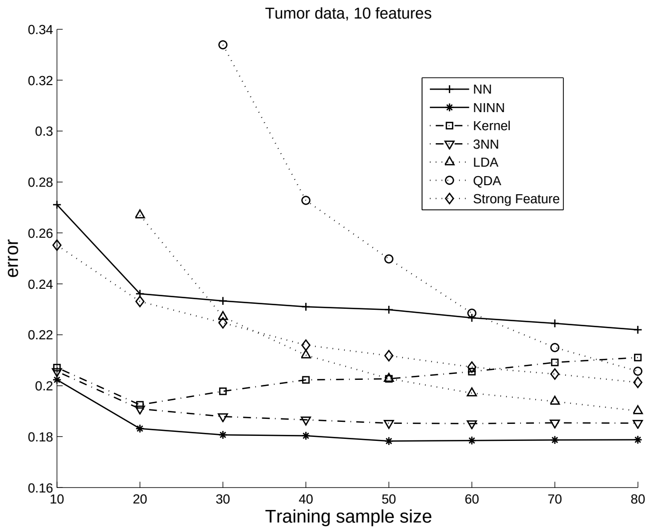

Figure 75: Classification error vs. training sample size for different classifiers.

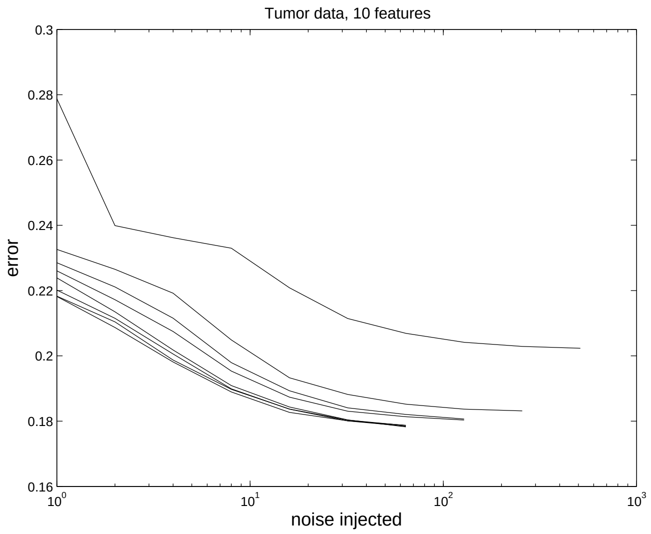

Figure 76: Classification results of noise injection.
